# Analysis in vivo using a new method, ARGO (Analysis of Red–Green Offset), reveals complexity and cell-type specificity in presynaptic turnover of synaptic vesicle protein Synaptogyrin/SNG-1

**DOI:** 10.1091/mbc.E25-09-0422

**Published:** 2026-06-10

**Authors:** Nikita Shiliaev, Manuel A. Alvarez, Ruiling Zhong, Sherlyn P. Wijaya, Sophie Baumberger, Claire E. Richardson

**Affiliations:** ^a^Department of Genetics, University of Wisconsin–Madison, Madison, WI 53706-1580; ^b^Wisconsin Shock Center of Excellence in Basic Biology of Aging (WiNSC), University of Wisconsin–Madison, Madison, WI 53706-1580; Brandeis University

## Abstract

In long-lived cells such as neurons, proteostasis involves the regulated degradation and replacement of proteins to ensure their quality and appropriate abundance. Synaptic vesicle (SV) protein turnover in neurons is important for controlling the SV pool size and sustaining appropriate levels of neurotransmission; yet, it is incompletely understood, partly due to limited tools for quantifying protein turnover in vivo. We present ARGO (Analysis of Red–Green Offset), a fully genetically encoded ratiometric fluorescence imaging method that visualizes and quantifies protein turnover with subcellular resolution in vivo. ARGO is inexpensive, modular, and scalable for use in genetically tractable experimental organisms. Using ARGO, we examine the turnover of Synaptogyrin/SNG-1, an evolutionarily conserved, integral SV protein, in *Caenorhabditis elegans* neurons. We show that the SNG-1 turnover rate is consistent across presynapses within a single neuron but varies between neuron classes. Notably, we find SNG-1 can exist in two distinct, nonintermixing populations within each presynapse. Furthermore, we present an initial mutant analysis of *uba-1*, the sole E1 ubiquitin ligase in *C. elegans*, demonstrating ARGO's capability to detect slowed SNG-1 turnover even though steady-state SNG-1 abundance is unchanged compared with wild-type. These results provide new hints for the regulation of the SV pool size.

## Abstract (executive summary)

- In long-lived cells, a protein's rates of synthesis and degradation together determine its abundance, yet regulation of protein turnover is largely unknown due to the lack of simple methods for in vivo quantification.
- Using *Caenorhabditis elegans*, the authors develop ARGO, a genetically encoded, microscopy approach that quantifies a protein's turnover with subcellular resolution. Results suggest that synaptic vesicle (SV) protein Synaptogyrin/SNG-1 can partition into two presynaptic pools with distinct half-lives.
- This provides a powerful tool to study protein turnover, reveals an unexpected complexity in SNG-1 turnover, and lays the groundwork for future investigations of SV protein compartmentalization, sorting, and degradation.

## INTRODUCTION

For neurons and other long-lived cells, maintenance of cellular function requires maintenance of proteostasis. Part of proteostasis is the continuous turnover and replacement of most proteins, which is proposed to generate multiple benefits; these include promoting stability in protein levels despite stochastic variations and errors in gene expression, removing damaged proteins, limiting accumulation of protein damage, and regulating protein abundance (Morimoto and Cuervo, 2014; Reddien, 2024). The importance of this continuous turnover is highlighted by the consequence of its failure: declining proteostasis, including the accumulation of misfolded and damaged proteins, is a hallmark of aging and a central driver of neurodegenerative disease (Hipp *et al.*, 2019; López-Otín *et al.*, 2023). Neurons’ complex morphology presents a spatial challenge for turnover of synaptic proteins, as most protein degradation machinery resides in the neuron cell body (Winckler *et al.*, 2018). However, homeostatic protein degradation remains incompletely understood for most proteins in any cell type. An obstacle to understanding this is the lack of easy and robust methods to interrogate protein half-life in vivo with high spatial and temporal resolution.

Several methods can be used to quantify homeostatic protein turnover. Pulse–chase labeling with heavy-isotope-containing amino acids followed by bulk proteomics to measure reduction in labeled protein over time has the strength of simultaneously measuring the half-life of thousands of proteins (Zhou, 2004). This method is laborious, though, especially in vivo, and the ability to measure protein half-life with cellular or subcellular resolution is limited since all of an animal's cells are labeled. Fluorescence microscopy-based approaches enable the assessment of an individual protein-of-interest's turnover with high resolution. This has been achieved using photoconvertible fluorescent proteins, which require calibration of the photoconversion to minimize phototoxicity in the pulse (Zhang *et al.*, 2007). Another strategy is to label the protein-of-interest with a self-labeling tag, most commonly SNAP-tag or HaloTag, provide a “pulse” of fluorescent ligand, then image turnover with fluorescence microscopy during the “chase” (Bojkowska *et al.*, 2011). Such experiments contributed evidence that, in cultured hippocampal neurons, synaptic vesicle (SV) proteins function in SV cycling for less than half of their total half-life, and, once decommissioned from SV cycling, they continue to reside at the presynapse (Truckenbrodt *et al.*, 2018). Considering those results, one might speculate that the management of SV protein turnover as a whole, including the sorting out of the functional pool, is especially stringent. Recently, this method was used to visualize and quantify the homeostatic degradation of postsynaptic protein PSD95 in the mouse brain using PSD95-HaloTag (Bulovaite *et al.*, 2022). This was the first time homeostatic protein degradation of any neuronal protein has been quantified with notable spatial resolution in vivo, and the results indicated that PSD95 turnover depends on animal age, region of the brain, and subcellular region within individual neurons (Bulovaite *et al.*, 2022). Such variation is consistent with the notion that synaptic protein turnover is highly regulated and may play an important role in specifying differences in synaptic function across different neuron types.

Importantly, comparing protein half-life between multiple separate studies that used the heavy-isotope-pulse-plus-proteomics approach shows that cultured neurons have dramatically shorter protein half-lives overall than neurons in vivo (Price *et al.*, 2010; Cohen *et al.*, 2013; Visscher *et al.*, 2016; Dörrbaum *et al.*, 2018; Fornasiero *et al.*, 2018; Heo *et al.*, 2018; Mathieson *et al.*, 2018; Kluever *et al.*, 2022). This indicates that there is a difference in the regulation of protein degradation in an adult animal's functioning nervous system versus in immature, cultured neurons. This could be due to a variety of factors, including neuron age, neuron morphology, or cell-extrinsic environment; nevertheless, it highlights the value of studying protein turnover in vivo.

Here, we developed a new method to visualize the turnover of a protein of interest with high spatial and temporal resolution in vivo using fluorescence microscopy. We call this method ARGO (Analysis of Red–Green Offset), drawing an analogy to the Theseus Ship Paradox, which ponders how identity persists even as components are gradually replaced over time. This method could, in theory, be applied to any protein-of-interest for which the addition of a fluorescent tag does not impact localization or function.

We used ARGO to begin to directly analyze the turnover of SV proteins in adult neurons in vivo. SV proteins are of particular interest because the abundance, composition, and functionality of SV pools at each presynapse contribute to determining the amount of neurotransmission. Furthermore, measuring their turnover provides an opportunity to test whether SV pools are maintained as spatially or functionally distinct compartments within individual neurons, as nonuniform intracellular turnover would imply such distinctions. We chose Synaptogyrin/SNG-1 as our first protein of interest because it is an evolutionarily conserved transmembrane SV protein, it is present on most SVs, and it regulates SV cycling (Abraham *et al.*, 2011; Raja *et al.*, 2019). These features make SNG-1 an informative starting point for establishing ARGO and for probing principles of SV protein turnover. As it is an integral membrane protein, it is expected to be degraded via endosomal sorting to lysosomal compartments.

Indeed, prior research mainly using assessment of steady-state SV protein localization combined with genetic analyses has led to the model that SV proteins are sorted into late endosomes and/or autophagosomes at the synapse, then transported to the cell body for degradation within lysosomal compartments (Uytterhoeven *et al.*, 2011; Waites *et al.*, 2013; Fernandes *et al.*, 2014; Sheehan *et al.*, 2016; Okerlund *et al.*, 2017). Interestingly, though, an imaging strategy to assess steady-state SV protein degradation during brain development in *D. melanogaster* found evidence that at least some SV protein is degraded in an axonal degradative “hub” (Jin *et al.*, 2018). Another aspect under investigation is how the rate of SV protein degradation is regulated, with evidence pointing to both surveillance and/or sorting for degradation as rate-limiting steps (Fernandes *et al.*, 2014; Sheehan *et al.*, 2016; Okerlund *et al.*, 2017; Sheehan and Waites, 2019; Hoffmann-Conaway *et al.*, 2020; Birdsall *et al.*, 2022). A more direct observation of SV protein turnover will potentiate a deeper understanding of this process.

## RESULTS

### Strategy and validation of SNG-1::ARGO

**FIGURE 1:**
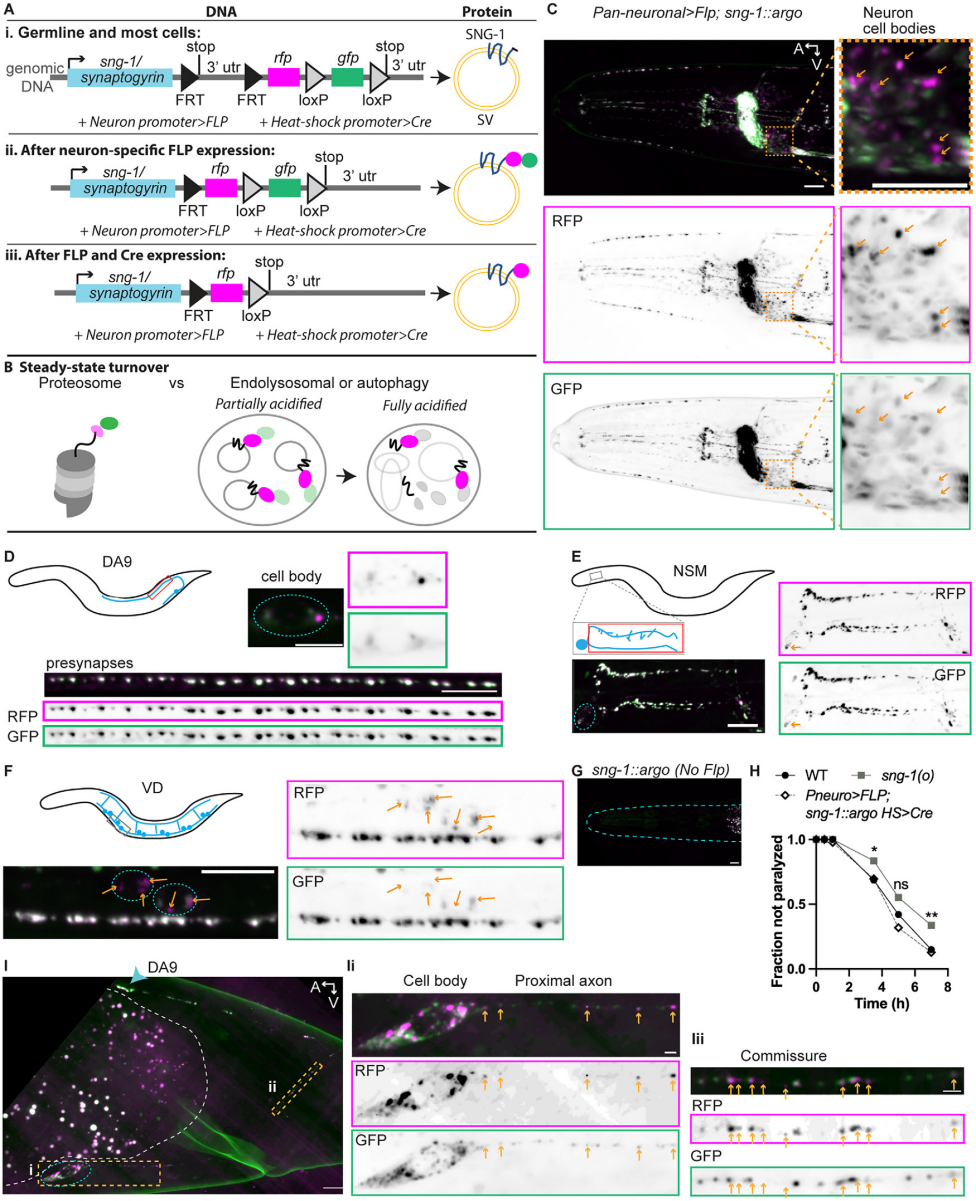
Steady-state SNG-1::ARGO fluorescence indicates that SNG-1 is degraded in lysosomal compartments in the cell body. (A and B) ARGO method overview. (Ai) The *argo* gene cassette is inserted into the gene-of-interest, ideally at the endogenous genomic locus. The strain also contains transgenes encoding neuron-specific FLP (Flippase) and a mechanism for inducible Cre expression. In this case, we tagged *sng-1* at the genomic locus using CRISPR/Cas9 and used a heat-shock promoter to induce Cre expression. (right) The protein-of-interest, in this case SNG-1, will be expressed with a short additional amino acid tag, which is encoded by the first FRT sequence, in every cell. (Aii) Neuron-specific promoter drives FLP expression shortly after neuron birth. These cells “turn on” the ARGO tag. (Aiii) (left) A pulse of Cre expression removes *gfp* from the *argo* gene cassette. This “activates” the ARGO. (right) All newly synthesized SNG-1 will be tagged with RFP. Therefore, RFP fluorescence reports total SNG-1 protein, whereas GFP reports “old” SNG-1. (B) Steady-state imaging provides clues as to how, and sometimes where, a protein-of-interest is degraded. For ARGO-tagged proteins that are degraded in lysosomal compartments, proteins that have been sorted for degradation will show stronger RFP versus GFP fluorescence, as GFP is quenched by the acidic environment of the endolysosomal and/or autolysosomal lumen. (C) Representative image of the head of an animal with the *sng-1:Iargo* allele in which the ARGO tag is flipped-on by a pan-neuronally expressed FLP, showing colocalized RFP and GFP fluorescence at presynapses throughout the nervous system. Zoomed-in views on the right show a region where neuron cell bodies reside. Arrows point to endosomes with relatively brighter RFP versus GFP intensity; scale bar, 10 µm (D–F) SNG-1::ARGO Flipped-on in individual neurons. Cartoons show neuron morphology (blue) within the animal (black). The red box shows where presynapses are located (D and E), gray boxes indicate the region imaged (E and F), and teal dashed ovals encircle neuron cell bodies (D and F). The VD neurons (F) make a string of en passant synapses along the ventral nerve cord. (G) In the absence of FLP expression, there is no detectable SNG-1:: ARGO fluorescence. The teal dashed curve outlines the nose of the animal. (H) Sensitivity to 0.5 mM aldicarb in Day 1 adults (A1) (ns: not significant, *P < 0.05, **P < 0.01, *χ*^2^ test with Bonferroni correction, *n* = 90 animals per genotype summed across three separate experiments). (I) SNG-1::ARGO in the DA9 neuron imaged with Super Resolution spinning disk confocal microscopy with deconvolution. The area inside the dashed white line contains autofluorescence from the intestine. The teal dashed oval encircles the neuron cell body; the teal arrowhead points to the most proximal presynapse. Note that transport packets containing SNG-1::ARGO are visible throughout the axon between the cell body and the proximal synapse; scale bar, 5 µm. (li–ii) Transport packets with brighter RFP versus GFP fluorescence are indicated with orange arrows; scale bar, 1 µm.

To enable the analysis of protein turnover with subcellular resolution in vivo, we developed the ARGO method (Figure 1A). The ARGO construct cell-specifically, cotranslationally labels the protein-of-interest with both RFP and GFP in a tandem tag. Cell specificity is achieved using FLP/FRT, wherein the Flippase is expressed from cell-specific promoters. The steady-state GFP/RFP ratio of an ARGO-tagged protein reflects the mechanism of degradation. As GFP fluorescence is quenched in acidic environments, ARGO-tagged proteins that have been sorted for degradation within lysosomal compartments are detected as endosomes with stronger RFP than GFP fluorescence (Figure 1B). Alternatively, the absence of detectable lysosomal compartments suggests that the protein-of-interest is degraded by the proteasome, or not at all. Next, the ARGO “pulse” removes *gfp* from the *argo* gene cassette such that all newly produced molecules of the protein-of-interest are labeled with only RFP. The neuron is then periodically imaged with fluorescence confocal microscopy, and the GFP/RFP ratio is quantified for each fluorescent punctum throughout the cell to determine the proportion of “old” versus “total” protein-of-interest, both spatially and temporally.

To study SV protein turnover, the *argo* cassette was inserted into the *sng-1* genomic locus such that the RFP::GFP tandem tag would be attached to the C-terminus of SNG-1, which resides on the cytoplasmic side of SVs (Figure 1A). Expression of Flippase from a pan-neuronal reporter shows SNG-1::ARGO dually labeled with RFP and GFP fluorescence throughout the nervous system (Figure 1C). Driving Flippase expression from neuron-specific promoters, we turned on SNG-1::ARGO expression in three individual neuron classes that represent a variety of morphologies and functions: the cholinergic motor neuron DA9, the two bilaterally symmetric serotonergic NSM neurons, and the GABAergic motor neurons VD/DD (Figure 1, D–F). Each of these neuron types elaborates multiple *en passant* synapses that can be visualized as individual puncta using fluorescence confocal microscopy (White *et al.*, 1986). The use of neuron-specific Flippase transgenes enables visualization of these individual presynapses, to which SNG-1::ARGO localizes as expected, with co-localized fluorescence from RFP and GFP (Figure 1, D–F) (Schwartz and Jorgensen, 2016; Muñoz-Jiménez *et al.*, 2017). In the absence of Flippase, no fluorescence is observed from the *sng-1::argo* allele (Figure 1G).

For the ARGO method to provide meaningful information about protein turnover, it must not impact the function of the protein-of-interest. This is because each of the facets of a protein's function, including folding, localization, and activity, is a factor that could be an input to regulate its turnover. For SNG-1::ARGO, proper localization to the presynapses is the first indicator that this allele is usable. Next, we assessed *sng-1* function. Synaptogyrin/SNG-1 regulates synaptic transmission from worms to mammals (Abraham *et al.*, 2011; Raja *et al.*, 2019). In *Caenorhabditis elegans*, loss of *sng-1* function causes resistance to the acetylcholinesterase inhibitor aldicarb, suggesting that the *sng-1(o)* mutant has reduced SV release compared with wild-type (Figure 1H) (Mahoney *et al.*, 2006; Abraham *et al.*, 2011). By contrast, pan-neuronally expressed SNG-1::ARGO has no discernable effect on aldicarb resistance (Figure 1H), suggesting that the SNG-1::ARGO protein is functional. Together, these results suggest that SNG-1::ARGO is likely a valid tool for investigating SNG-1 biology.

To generate the pulse that activates ARGO, we excised *gfp* from the *argo* gene cassette using a heat-shock promoter to drive production of Cre recombinase upon a brief heat shock. This is the quickest and most robust method to induce gene expression in *C. elegans*. Indeed, by assessing fluorescence in non–heat-shocked animals and in animals several days post–heat-shock, we observed that this method efficiently activated SNG-1::ARGO in each of the three neuron types we examined, with little ectopic excision of *gfp* in the absence of heat shock (Table 1; Supplemental Figure S1A). Though it is practical, a drawback to this approach is that heat shock causes increased production of HSF-1-regulated genes, including chaperones, which will impact proteostasis. To assess whether this is likely to have a substantial impact on SNG-1 turnover dynamics or mechanisms, we assessed whether *sng-1* mRNA or protein abundance is altered by the heat shock pulse. We reasoned that, if neither the rate of SNG-1 production nor the steady-state abundance of SNG-1 is impacted by the heat shock, then it is unlikely that the degradation is altered. We observe no significant difference in *sng-1* mRNA levels either 5 h or 1 d after heat-shock (Supplemental Figure S1B). To assess steady-state SNG-1 protein abundance, we quantified average presynaptic SNG-1::ARGO RFP intensity and number of synapses in the DA9 neuron. There was no significant difference 4 h, 1 d, or 2 d after heat shock compared with control (Supplemental Figure S1, C and D). We detect an increase in the presynaptic SNG-1::ARGO GFP/RFP ratio immediately following heat-shock that is not detectable by 4 h or 1 day post-heat shock (Supplemental Figure S1E). That timing is expected to be too fast to reflect a change in *sng-1* gene expression; it is therefore likely to reflect a change in localization, such as decreased SV cycling or trafficking. Still, the difference is resolved by 4 h post-heat shock, and that the first post-heat shock imaging timepoint used in turnover experiments is 1 d. Together, these data indicate that SNG-1::ARGO turnover is unlikely to be substantially impacted by the heat shock pulse. Concerns about a potential subtle impact are mitigated by the fact that all turnover comparisons were made between cohorts that received the same heat shock.

**TABLE 1:** Specificity and efficacy of SNG-1::ARGO activation by heat-shock–promoter driving *Cre* recombinase.

Specificity		
Neuron	Age scored (d of adulthood)	GFP not recombined without heat shock (*n*)
DA9	≥4	95% (146)
NSM	≥6	100% (43)
VD2	≥5	99% (88)
**Efficacy**		
**Neuron**	**Age heat-shocked (d of adulthood)**	**GFP recombined after heat-shock (*n*)**
DA9	0	97.3% (108)
DA9	2	94% (65)
NSM	2	100% (55)
VD2	2	97% (38)

*Note:* Parameters were assessed in strains carrying sng-1(syb3140car2[argo]) for DA9 and NSM and sng-1(syb3140[unstable argo]) for VD2. All strains carried an individual neuron-specific flippase (one per strain), and an integrated transgene composed of a heat-shock promoter driving Cre recombinase (heSi160). Specificity means how often the GFP was not excised in the absence of heat-shock, and Efficacy means how often the GFP was excised upon heat-shock of 1 h at 34°C.

Experimentally, the ARGO pulse is a 1-h heat shock at 34°C, but the pulse in terms of generating a Time = 0 for turnover experiments requires production of Cre, Cre-mediated removal of the *gfp* gene, and degradation of the mRNA molecules encoding *sng-1::rfp::gfp.* We assessed the effective timing of the pulse by imaging presynapses 4 h post-heat shock (Supplemental Figure S1, F–L). Whereas we detect no change in SNG-1::ARGO GFP/RFP in a heat-shocked strain lacking the *HS>Cre* transgene, there was a significant decrease in the GFP/RFP ratio in five of the six neuron-by-animal-age conditions tested (Supplemental Figure S1, F–L). In addition, the abundance of Cre-recombined *sng-1::argo* alleles in the genome peaked by 4 h post-heat shock (Supplemental Figure S1M). Therefore, the ARGO pulse effectively takes on the order of several hours, and the ARGO method systematically overestimates protein half-life by that much. For comparison, the mean half-life of the *C. elegans* proteome is estimated to be on the order of a day or two (Depuydt *et al.*, 2016; Visscher *et al.*, 2016).

### SNG-1 is likely sorted for degradation at the presynapse and degraded in the cell body

Assessing steady-state SNG-1::ARGO, we observed that there are fluorescent puncta within the neuron cell bodies that show relatively stronger RFP intensity compared with GFP intensity (Figure 1, C–F). Super-resolution microscopy resolved two distinct populations of SNG-1::ARGO-labeled endosomes in the neuron cell body: GFP-brighter endosomes, which are likely newly synthesized and trafficking endosomes, and RFP-brighter endosomes, which are likely SNG-1 within acidic lysosomal compartments (Figure 1I). To test this interpretation, we assessed colocalization of SNG-1::ARGO with NUC-1::BFP, a lysosomal luminal protein (Sun *et al.*, 2020). Indeed, NUC-1::BFP colocalizes with the RFP-brighter SNG-1::ARGO endosomes but not with the GFP-brighter SNG-1::ARGO endosomes (Supplemental Figure S2). Furthermore, transport packets of both the GFP-brighter and RFP-brighter flavors are present along the proximal axon and commissure (Figure 1I). We observed co-localization between SNG-1::ARGO and BFP::RAB-3, an SV protein, in the soma and at the presynapses (Supplemental Figure S3). The SNG-1::ARGO GFP intensity correlated with the BFP::RAB-3 intensity; both signals are expected to correlate with SV number (Supplemental Figure S3). To confirm the specificity of the SNG-1::ARGO fluorescence signals, we performed RNA interference (RNAi) against *sng-1*. In the knockdown animals, the fluorescence previously observed across all subcellular domains was abolished (Supplemental Figure S4), validating that the detected signals arise from endogenous SNG-1::ARGO expression. Together, these data suggest that SNG-1::ARGO is trafficked retrogradely from the presynapses to the cell body in acidified lysosomal compartments for degradation (Figure 1I).

**FIGURE 2:**
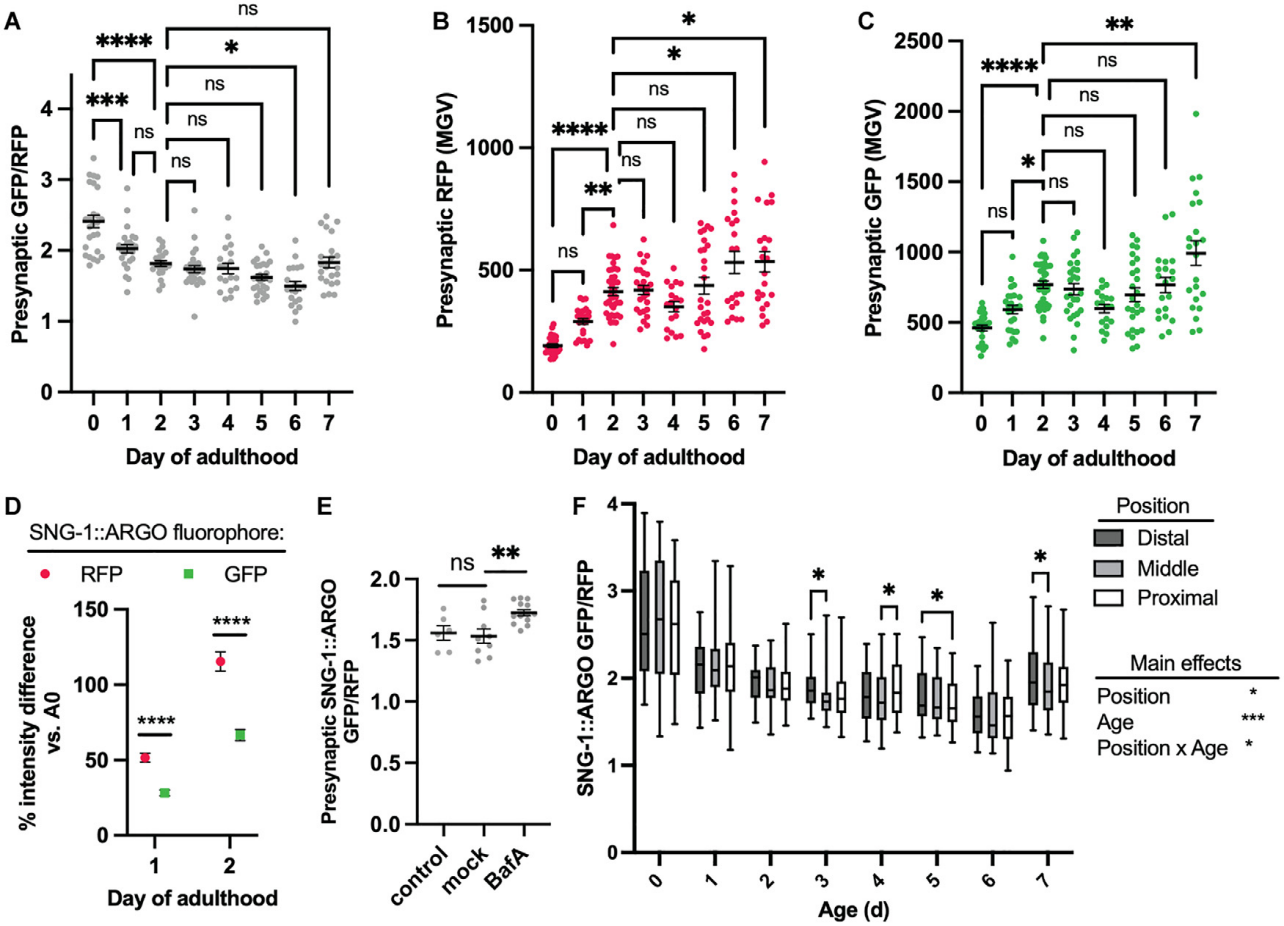
Presynaptic SNG-1::ARGO shows a decline in steady-state GFP/RFP fluorescence, indicative of accumulation within acidic compartments, during early adulthood in the DA9 neuron. (A–C) Average presynaptic RFP/GFP ratio (A), and the underlying intensity data for RFP (B) and GFP (C) from SNG-1::ARGO. Data points are each the mean of all presynapses from a single neuron. Here and elsewhere, unless otherwise noted, mean ± SEM are plotted. (D) The percentage of change in average presynaptic RFP and GFP intensity from day 0 (A0) through day 2 (A2) of adulthood, calculated from the data in B and C. (E) Presynaptic SNG-1:ARGO GFP/RFP in A3 animals injected with Bafilomycin A (BafA), vehicle (“mock”), or uninjected (“control”), quantified and displayed as in A. (F) SNG-1: ARGO presynaptic GFP/RFP by presynapse position along the axon. ns, not significant, *P < 0.05, **P < 0.01, ***P < 0.001, ****P < 0.0001, one-way ANOVA (A–C and E) or two-way ANOVA (D and F) with Tukey posttest.

We measured the steady-state SNG-1::ARGO GFP/RFP ratio at each presynapse per neuron in the DA9 neuron from day 0 (L4 larval stage, A0) to 7 of adulthood (A7) (Figure 2A). For comparison, the mean adult lifespan is ∼15 d (Gems and Riddle, 2000). Note that GFP has a higher quantum yield than RFP and is consistently brighter in vivo, so a ratio greater than 1 was expected (Rodriguez *et al.*, 2017). Indeed, the mean presynaptic SNG-1::ARGO GFP/RFP ratio at A0 was >2 (Figure 2A). Mean presynaptic SNG-1::ARGO GFP/RFP declined between A0 and A2, then remained fairly stable, with a trend for progressive slight decrease that was resolved at A7 (Figure 2A).

To better understand the change in ratio between A0 to A2, we examined the RFP and GFP intensities underlying the ratiometric data (Figure 2, B and C). *C. elegans* body size increases between A0 and A2, and the DA9 synapses likewise increase in both number and size based on the RFP intensity (Figure 2, B and D; Supplemental Figure S1D). The average steady-state (no pulse) SNG-1::ARGO GFP fluorescence intensity per synapse likewise increases, but by only half as much, underlying the decrease in the presynaptic SNG-1::ARGO GFP/RFP ratio (Figure 2, C and D).

We hypothesized that 1) SNG-1::ARGO is sorted into acidified compartments, likely late endosomes, at the presynapse, which partially quenches the GFP fluorescence, and 2) the steady-state proportion of presynaptic SNG-1::ARGO that resides within acidified compartments increases in early adulthood. To test this hypothesis, we injected mid-adult animals with Bafilomycin A, which inhibits the V-ATPase that acidifies lysosomes and late endosomes. Indeed, animals injected with Bafilomycin A showed an increase in the presynaptic SNG-1::ARGO GFP/RFP ratio 3 h postinjection compared with control and vehicle-injected animals (Figure 2E). Furthermore, we have shown that injecting animals with concanamycin A, which also blocks the V-ATPase, increased the SNG-1::ARGO GFP/RFP ratio at both the presynapse and the neuron soma within hours (Zhong and Richardson, 2025). Consistent with this interpretation, late endosomes and autolysosomes, which both acquire acidified lumens, localize to presynapses (Ceccarelli *et al.*, 1973; LaVail and LaVail, 1975; Grill *et al.*, 2007; Binotti *et al.*, 2014; Soukup *et al.*, 2016; Stavoe *et al.*, 2016; Neisch *et al.*, 2017). Still, neither the Bafilomycin A injection shown here nor the concanamycin A injection in our prior work fully restore the SNG-1::ARGO GFP/RFP to the expected “unquenched” ratio; this may reflects incomplete efficiency of the drug treatment (see Materials and Methods section), but we cannot rule out the possibility that an additional factor, aside from GFP quenching in acidic endosomal compartments, contributes to the changing SNG-1::ARGO GFP/RFP ratio.

Taken together, these data suggest that SNG-1::ARGO is sorted for degradation into acidified compartments at the presynapse and then trafficked to the cell body to complete degradation. This aligns with the prevailing model for SV protein turnover, which was mainly based on more indirect evidence, including genetic analysis of steady-state abundance and the presence of MVBs and autophagosomes at presynapses. Of note, SNG-1 within partially acidified compartments is not necessarily destined for degradation; this notion is considered further below.

Next, we assessed whether the presynaptic SNG-1::ARGO GFP/RFP ratio is dependent on presynapse position. We binned the presynapses as “proximal,” “middle,” or “distal” along the axon and compared the steady-state GFP/RFP for each day (Figure 2F). There was no consistent relationship between synapse position and ratio (Figure 2F).

### Quantification of presynaptic SNG-1::ARGO turnover in the DA9 neuron

**FIGURE 3:**
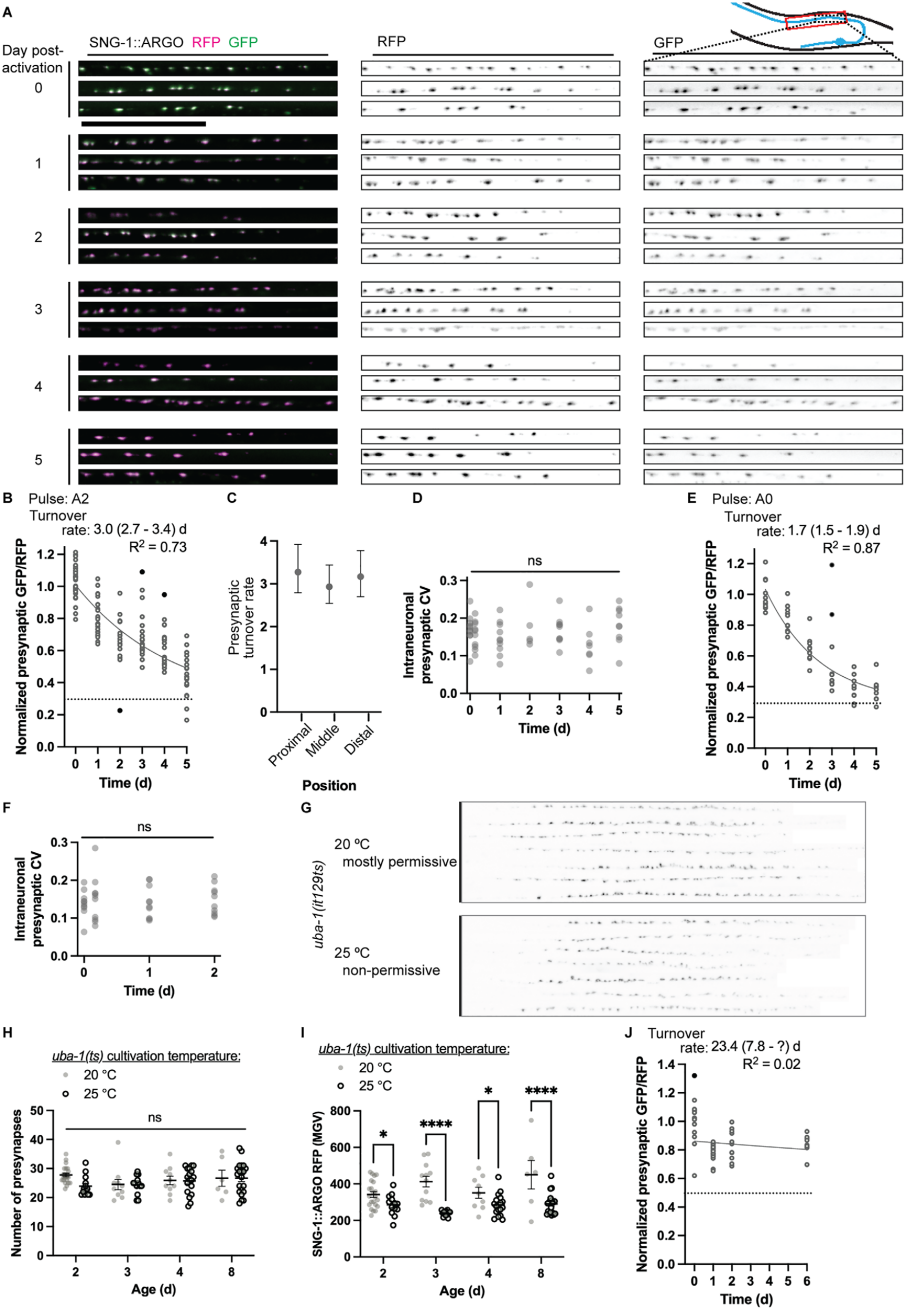
Quantification of SNG-1::ARGO turnover in the DA9 neuron. (A and B) Example images (A) and quantification (B) of SNG-1::ARGO in DA9 presynapses from the turnover experiment with the pulse at A2. (A) Proximal synapses from three animals per timepoint (different cohorts of animals are imaged at each timepoint); scale bar, 30 µm. (B) One-phase exponential decay function fit to SNG-1::ARGO turnover data with the pulse at A2; each data point shows the average presynaptic SNG-1::ARGO GFP/RFP ratio from all the presynaptic puncta in a single neuron. The dashed black line shows the experimentally calculated background, which comes from autofluorescence. Outlier data points that were excluded from the function calculation are shown in black. (C) SNG-1::ARGO presynaptic half-life by proximal–distal position relative to the neuron cell body (mean ± 95% CI). (D) The average CV for all the presynaptic puncta within each neuron does not change at any chase timepoint compared with the pulse, suggesting that there is effectively a uniform SNG-1::ARGO turnover rate across all presynapses within one neuron. (E and F) From a turnover experiment with the pulse at A0, one-phase exponential decay function (E) and intraneuronal variance between synapses (F). (G–I) The *uba-1(it129ts)* mutant at the nonpermissive temperature, 25°C, shows no obvious defect in presynapse localization (G) or number (H), but reduced abundance of SNG-1::ARGO at each presynapse (I). Two-way ANOVA with Tukey posttest. (J and K) A turnover experiment started at A2 indicates that the SNG-1::ARGO presynaptic turnover is slowed in the *uba-1(it129ts)* mutant (J). ns: not significant, *P<0.05, ****P<0.0001.

Using the DA9 neuron, we pulsed SNG-1::ARGO at A2 and then imaged unique cohorts of animals daily for 5 d afterward, plus a cohort immediately before and after the activation (Figure 3, A and B; Supplemental Figure S1H). SNG-1::ARGO GFP/RFP ratios were normalized to the mean ratio from age-matched control (nonpulsed) cohorts imaged in parallel. This normalization was used for all SNG-1::ARGO turnover experiments that use the GFP/RFP ratio throughout this study so that the apparent rate of turnover is not impacted by any age-dependent GFP quenching within lysosomal compartments. Importantly, though, for SNG-1::ARGO and any ARGO-tagged protein degraded by the lysosome, the pulse–chase experiments quantify the rate at which the protein-of-interest is delivered to fully acidified lysosomal compartments, when the GFP fluorescence disappears. That rate is likely shorter than the physical half-life of the protein on the order of hours, though if lysosomal degradation is not working efficiently, the time difference between quenching and degradation could be longer (Barral *et al.*, 2022). From A2, presynapse size (based on SNG-1::RFP intensity) and presynapse number are fairly stable (Figure 2B; Supplemental Figure S1, A, C, and D). This suggests that the rate of SNG-1::ARGO production and delivery to the presynapse matches the rate of SNG-1::ARGO removal for degradation. Therefore, the declining SNG-1::ARGO GFP/RFP each day after the pulse is predominantly due to SNG-1 turnover. Fitting a single-phase exponential decay function to the data calculated an apparent presynaptic turnover rate of 3.0 d (95% CI, 2.7–3.4 d). We observed no impact of presynapse position relative to the neuron cell body on the turnover rate (Figure 3C). To assess whether some other feature of individual presynapses might regulate turnover rate within a neuron, we quantified the intraneuronal coefficient of variation (CV) in presynaptic SNG-1::ARGO GFP/RFP for each timepoint in the pulse–chase (Figure 3D). If the rate of SNG-1::ARGO turnover varies by presynapse, we expect to observe an increase in CV during the chase, especially at timepoints close to the pan-synaptic *t*_1/2_ for turnover, compared with *T* = 0. However, no change in CV was detected.

We performed a pulse–chase experiment with the DA9 SNG-1::ARGO starting at A0. From A0 to A2, the DA9 presynapses grow by over 100%, and additional presynapses are added, scaling with increasing animal size (Figure 2, B–D; Supplemental Figure S1D). This means that the rate of SNG-1::ARGO production plus delivery to the presynapse exceeds the rate of SNG-1 removal from the synapse for degradation during this time period. The apparent presynaptic SNG-1::ARGO turnover rate—1.7 d (95% CI, 1.5–1.9 d)—therefore reflects both the removal of old RFP::GFP-positive SNG-1::ARGO molecules from the synapse and the increase in total abundance of RFP-only SNG-1::ARGO at the presynapses (Figure 3E). The shorter apparent turnover rate with the pulse at A0 compared with A2 is likely due to this scaling as opposed to a change in the rate of SNG-1 degradation. Indeed, fitting one-phase exponential decay curves to both the A2 and A0 datasets using the GFP intensity rather than the GFP/RFP ratio, which approximately quantifies just the half-life of the pre-existing SNG-1 proteins, we observe no difference in apparent half-life between the two different pulse times (Supplemental Figure S5, A and B). With the pulse at A0, the calculated SNG-1 half-life is longer using mean GFP, which quantifies only degradation, compared with using the GFP/RFP ratio that quantifies degradation plus synapse growth. Also, considering that the presynaptic SNG-1::ARGO GFP fluorescence becomes partially quenched between A0 and A2 (Figure 2, A–E), the SNG-1 half-life calculated by mean GFP for A0 is an underestimate. By contrast, with the pulse at A2, the half-life calculated using mean GFP is not confounded by the steady-state increase in GFP quenching, and it is indistinguishable from that calculated using the GFP/RFP ratio, though the 95% CI is substantially larger. Indeed, using the GFP intensity instead of the GFP/RFP ratio to fit the exponential decay function results in substantially lower *R*^2^ values (>0.72 for ratiometric vs. <0.29 for GFP only) and wider 95% CIs (<0.8 d for ratiometric vs. >2.4 d for GFP only) for both experiments (Figure 3, B–D; Supplemental Figure S5, A and B). This demonstrates the utility of incorporating ratiometric quantification for turnover analysis.

To assess the validity of the SNG-1::ARGO turnover calculations, we generated two strains that express SNG-1 fused to the photoconvertible fluorophore Dendra2 in the DA9 neuron. Dendra2 irreversibly converts from green fluorescence to red fluorescence upon exposure to 405 nm light (Gurskaya *et al.*, 2006). In separate experiments, one with each strain, we pulsed animals at A0, and then we imaged unique cohorts of animals for 3 to 5 d afterward (Supplemental Figure S6). We quantified the mean fluorescence intensity at each presynapse in both the red channel and the green channel, and we calculated the turnover rate by fitting a one-phase exponential decay curve to the data, using the average red/(red+green) intensity value per worm. With this approach, the apparent half-life was 1.3 d (95% CI, 1.0–1.6 d) with the first transgenic line (Supplemental Figure S6B) and 1.7 d (95% CI, 1.4–2.2 d) with the second. This analysis pipeline is comparable to the SNG-1::ARGO turnover analysis at A0 (Figure 3E). Although this experiment used SNG-1::Dendra2 expressed of a neuron-specific promoter instead of from the endogenous locus, unlike SNG-1::ARGO, the calculated apparent half-life was similar between the Dendra2 and ARGO approaches.

Returning to the presynaptic scaling that occurs between A0 and A2, the SNG-1::ARGO pulse–chase experiment affords an opportunity to assess whether newly synthesized SNG-1::ARGO, which is tagged with only RFP, is allocated to the presynapses with any spatial pattern. Such a pattern could be generated by synapse position or activity (i.e., the amount of SV cycling), and it could be detected as an increase in the intraneuronal CV in presynaptic SNG-1::ARGO GFP/RFP ratio in the chase compared with the pulse. No such increase was detected, indicating that newly synthesized SNG-1 is evenly distributed throughout the neuron's presynapses (Figure 3F). This is consistent with the model that SV deposition at both newly forming and fully formed presynapses strongly depends on trafficking parameters, and continuous trafficking intermixes SVs between synapses (Staras *et al.*, 2010; Herzog *et al.*, 2011; Wu *et al.*, 2013; Maeder *et al.*, 2014; Lipton *et al.*, 2018).

### SNG-1::ARGO turnover is slowed in a *uba-1*/E1 Ubiquitin ligase loss-of-function mutant

As an initial mutant analysis using the ARGO method and a first step toward defining the mechanisms of SNG-1 turnover, we constructed a strain carrying the DA9 *sng-1::argo* alleles and a temperature-sensitive hypomorphic allele of *uba-1(it129ts),* which encodes the sole *C. elegans* E1 ubiquitin ligase (Kulkarni and Smith, 2008). Ubiquitination marks proteins for degradation via the proteasome, the endolysosomal system, and some types of autophagy (Foot *et al.*, 2017; Yin *et al.*, 2020). Animals cultivated at the nonpermissive temperature, 25°C, looked fairly wild-type in terms of presynaptic localization, organization, and number (Figure 3, G and H; Supplemental Figure S7, A–F). The *uba-1(it129ts)* mutants grown at the nonpermissive temperature showed a reduction in presynaptic SNG-1::ARGO RFP intensity compared with *uba-1(it129ts)* grown at the permissive temperature (Figure 3I) but not compared with wild-type grown in parallel at 25°C (Supplemental Figure S7, A–F). These data suggest that the accumulation of SNG-1::ARGO at the presynapse is mildly dependent on temperature but not dependent on *uba-1.*

In a turnover experiment with the pulse at A2, the *uba-1(it129ts)* mutant showed a presynaptic SNG-1::ARGO half-life of 23.4 d (95% CI, 7.8-[upper limit could not be calculated]) (Figure 3J, see also Supplemental Figure S5C). These data suggest that ubiquitination is likely required to promote SNG-1 degradation from the presynapse. Because the *uba-1(it129ts)* mutant is hypomorphic rather than amorphic, it is possible that UBA-1 is essential for all presynaptic SNG-1 turnover.

One model to explain the *uba-1(it129ts)* turnover result is that SNG-1 gets ubiquitinated and then sorted for degradation via ESCRT. If that were the case, we would expect the *uba-1(it129ts)* mutant to show an increased presynaptic SNG-1::ARGO GFP/RFP ratio compared with wild-type grown in parallel. However, we detect no difference in the presynaptic SNG-1::ARGO GFP/RFP ratio in the *uba-1(it129ts)* mutant compared with wild-type (Supplemental Figure S7F). In the soma, though, the SNG-1::ARGO endosomes with the lowest GFP/RFP ratio are missing in the *uba-1(it129ts)* mutant, suggestive of defective SNG-1 degradation (Supplemental Figure S7, I–L). This observation supports an alternate hypothesis in which presynaptic SNG-1::ARGO turnover is regulated indirectly by ubiquitination of other protein(s) in the neuron.

Whereas transmembrane proteins like SNG-1 are degraded by the lysosome, ubiquitination is most prominently required for protein degradation via the proteasome (Jackson and Hewitt, 2016). To test the possibility that *uba-1* promotes SNG-1::ARGO degradation indirectly, we assessed the impact of the proteasome inhibitor bortezomib on SNG-1::ARGO. We treated DA9 SNG-1::ARGO animals with bortezomib from A0, treated them with the heat shock-pulse at A2 alongside the untreated control animals, and imaged the presynapses at A5 (Supplemental Figure S8). Steady-state SNG-1::ARGO abundance, assessed by RFP, was not detectably impacted by bortezomib (Supplemental Figure S8B). The steady-state SNG-1::ARGO GFP/RFP ratio was also not affected by bortezomib, suggestive of normal presynaptic sorting for degradation (Supplemental Figure S8C). However, the bortezomib treatment caused a reduction in SNG-1::ARGO turnover, indicating that proteasomal degradation is required for normal SNG-1 turnover (Supplemental Figure S7C). It is unclear from our data how this interaction works, though several models are considered in the Discussion.

Of note, one might predict that a reduction in protein degradation during routine turnover could be revealed with steady-state imaging as an increased abundance of the protein-of-interest, either at its typical subcellular localization and/or elsewhere within the cell. However, we observe no increased accumulation of SNG-1::ARGO in the *uba-1(it129ts)* mutant compared with wild-type in the presynapse, the soma, or the dendrite (Supplemental Figure S7). These results highlight the utility of quantifying turnover.

### Steady-state presynaptic SNG-1::ARGO varies with neuron identity

We assessed steady-state presynaptic SNG-1::ARGO GFP/RFP in the NSM and VD2 neurons. First, we compared the intraneuronal CV across all presynaptic puncta of cohorts of DA9, NSM, and VD2 neurons at A2. If any neuron type showed a notably higher interneuronal CV, it could indicate that there is differential regulation in the sorting of SNG-1::ARGO into acidic compartments within individual presynapses. However, the NSM interneuronal CV was similar to that of DA9, and the CVs within VD2 neurons were low (Supplemental Figure S5D).

For the NSM neuron, we assessed steady-state SNG-1::ARGO across early adulthood and performed a turnover experiment with the pulse at A2. The SNG-1::ARGO steady-state and turnover characteristics generally were similar for NSM to those of the DA9 neuron (Supplemental Figure S5, E–K). The NSM neuron, however, showed a stronger steady-state position effect, with a trend that the more proximal synapses had a lower SNG-1::ARGO GFP/RFP ratio compared with the more distal synapses (Supplemental Figure S5I). Perhaps this is indicative of an increased density of SNG-1–containing lysosomal compartments closer to the cell body, which could arise due to SNG-1 retrograde trafficking in acidified compartments.

The VD2 neuron shows fairly stable SNG-1::ARGO fluorescence from A2 to A7 and no significant effects of synapse position relative to the soma on synapse fluorescence (Supplemental Figure S5, L–O).

### Biphasic turnover of SNG-1::ARGO within each VD2 presynapse

**FIGURE 4:**
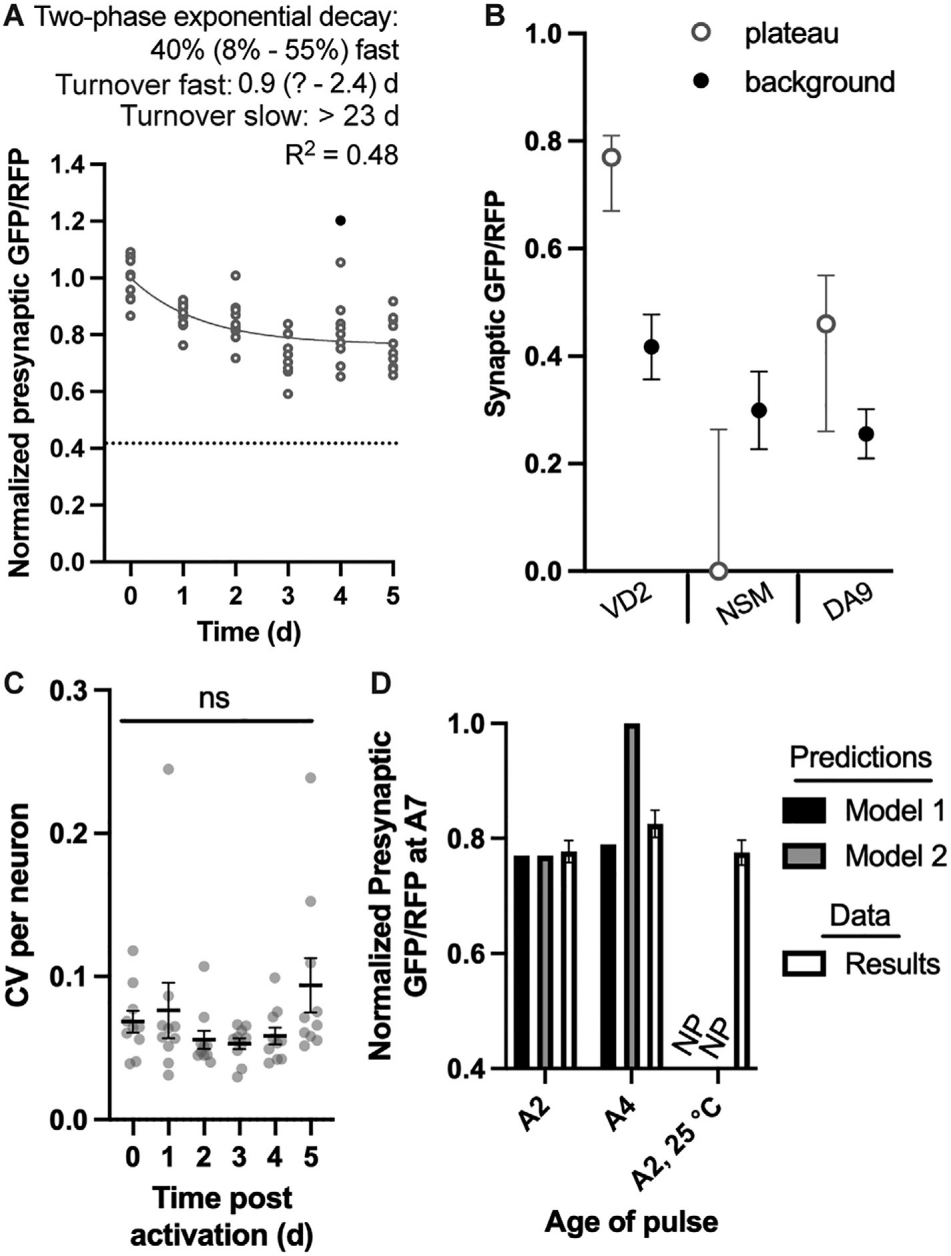
Biphasic SNG-1::ARGO turnover in the VD2 neuron. (A) SNG-1::ARGO turnover experiment in the VD2 neuron with the pulse at A2. Best-fit parameters and curve for a two-phase exponential decay function are shown, as this model was significantly favored over a one-phase exponential decay. (B) Comparison of the one-phase exponential decay function plateau (best-fit value and 95% CI) for each neuron identity with the pulse at A2. Data are from the experiments shown in 5A (VD2), S4I (NSM), and 3B (DA9). (C) Intraneuronal variance between individual presynaptic puncta during the VD2 turnover experiment. Each data point shows the CV for all the presynaptic puncta from one neuron. (D) Predicted presynaptic GFP/RFP values from the two models, qualitatively compared with results from a separate experiment in which animals with VD2 neuron SNG-1::ARGO were pulsed at different ages/conditions and imaged at A7. NP, no prediction; ns, not significant.

In the VD2 neuron with SNG-1::ARGO activation at A2, a one-phase exponential decay curve does not fit the data as well as it does for the DA9 and NSM neurons (VD2 *R*^2^ = 0.41, DA9 *R*^2^ = 0.73, NSM *R*^2^ = 0.63) (Figures 3B and 4A; Supplemental Figure S4J). A two-phase exponential decay curve fits the VD2 data better (*R*^2^ = 0.48), and it is apparent that the curve plateaus above the calculated background (Figure 4A). Indeed, according to the best-fit parameters for the two-phase curve, 40% of the SNG-1::ARGO molecules turn over with a *t*_1/2_ of about 0.9 d, and the other 60% turn over very slowly (*t*_1/2_ > 23 d) (Figure 4A). We considered whether this two-phase turnover could be due to nonspecific or ineffective Cre-mediated recombination specifically for the VD2 neuron, that is, either some VD2 neurons would fail to recombine one or both *gfp* alleles after the pulse and/or some VD2 neurons would recombine one or both alleles of *gfp* during steady-state imaging. To assess this idea, we evaluated the interneuronal CV for each neuron-by-age-by-treatment (Supplemental Figure S9). If the VD2 neuron had inefficient and/or nonspecific removal of *gfp,* we would expect to observe a higher CV in the VD2 neuron during the pulse–chase experiment and/or steady-state imaging compared with the DA9 and NSM neurons. This trend was not observed (Supplemental Figure S9).

As an alternate approach to assess whether the one-phase exponential decay function can be ruled out as a good descriptor of VD2 SNG-1::ARGO turnover, we fit a one-phase exponential decay function to the data with an unconstrained plateau. The best-fit plateau was significantly higher than the calculated background, and the 95% CIs from the two calculations did not overlap (Figure 4B). By the same analysis, the best-fit plateau and 95% CI for the NSM and DA9 neurons overlap with that of the calculated backgrounds (Figure 4B).

We assessed whether the two-phase function in the VD2 neuron arose from distinct turnover kinetics at spatially separate presynaptic puncta, that is, some presynaptic puncta show turnover with a 0.9-d *t*_1/2_ for turnover, while other presynaptic puncta within the same neuron show effectively no turnover. To do this, we calculated the CV between all the presynaptic SNG-1::ARGO GFP/RFP ratios within each neuron across each timepoint of the turnover experiment (Figure 4C). We found no significant increase in the average CV at any day postactivation compared with *T* = 0 (Figure 4C). These data support the model that each presynaptic punctum within an individual VD2 neuron exhibits the biphasic turnover.

We considered two models to explain the biphasic turnover in the VD2 presynapses. In Model 1, there are two chronically distinct populations of SNG-1::ARGO within each presynapse, one with a relatively fast turnover and the other with an unappreciable turnover. In Model 2, the mechanisms that promote SNG-1 turnover are shut off or become dysfunctional as the animal ages, at an age that is spanned by the turnover experiment. Model 1 predicts that the proportion of SNG-1 with an unappreciable turnover will be the same regardless of animal age at which the SNG-1::ARGO is activated (Figure 4D). By contrast, Model 2 predicts that the proportion of SNG-1 with an unappreciable turnover will be lower when SNG-1::ARGO is activated in younger animals and higher when it is activated in older animals (Figure 4D) (see Methods). To test these models, we performed an experiment in which we activated the VD2 SNG-1::ARGO at A2 versus A4 (days 2 and 4 of adulthood, respectively) and imaged both groups at A7. The A2 group recapitulated the result from the full turnover experiment, and the A4 group showed a similar amount of turnover as the A2 group, consistent with Model 1 (Figure 4D). We also assessed the presynaptic GFP/RFP ratio in animals that were pulsed at A2, then cultivated at 25°C instead of the standard 20°C until imaging at A7. Animals age faster, have a shorter lifespan, and exhibit compromised proteostasis at 25°C; still, the presynaptic SNG-1::ARGO GFP/RFP was about the same (Figure 4D); therefore, these results also align better with Model 1 than Model 2.

### Evidence for endocytic sorting of SNG-1 in presynapse remodeling

**FIGURE 5:**
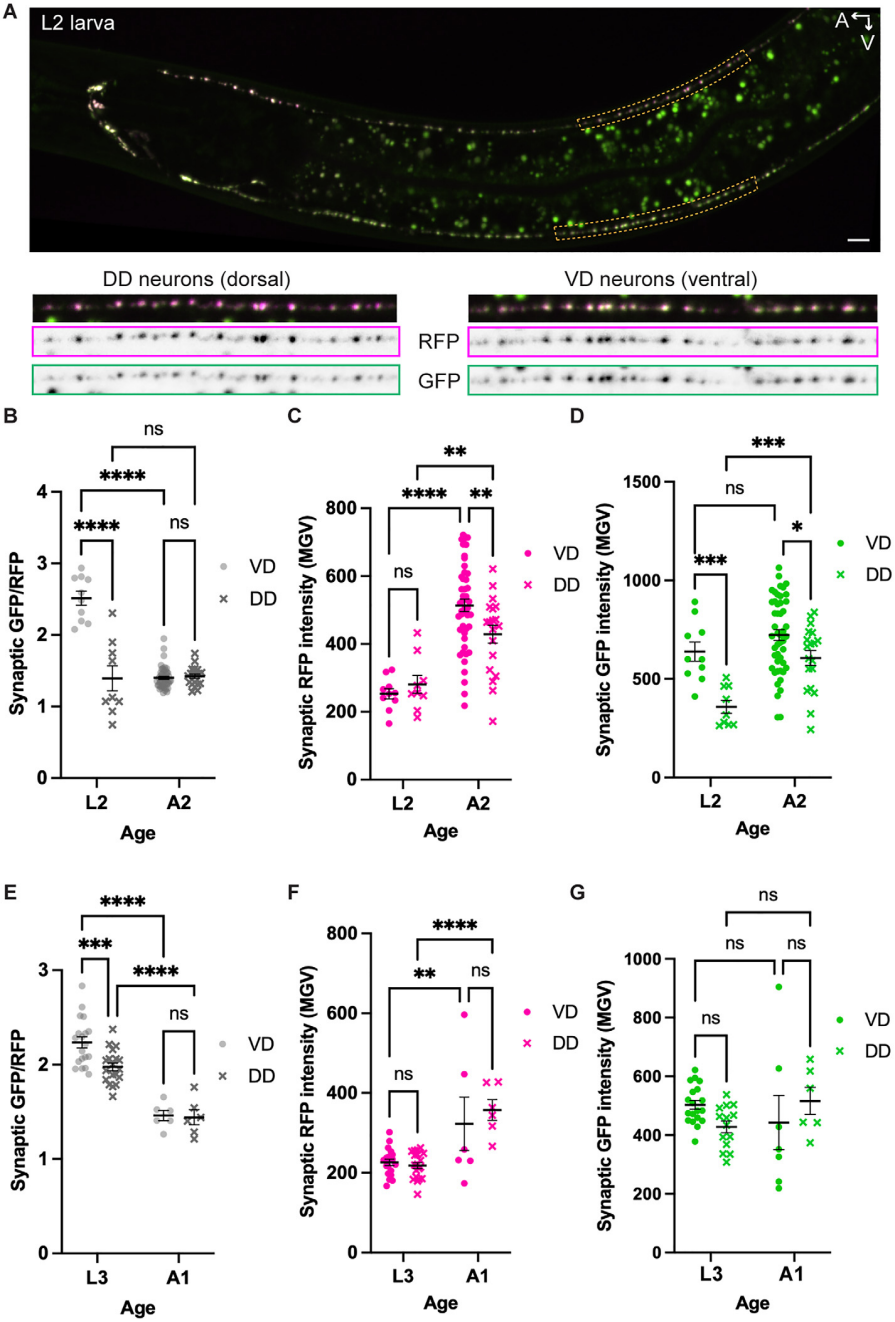
SNG-1::ARGO steady-state GFP/RFP ratio is low in the newly remodeled DD presynapses. (A) Representative image of SNG-1::ARGO in an L2 larvae, in which a lower GFP/RFP ratio in the DD neurons compared with the VD neurons is apparent. (B–G) Comparison of presynaptic SNG-1::ARGO GFP/RFP ratio (B and E) and raw fluorescence intensities (C–D and F–G) in the VD versus DD neurons shows a difference at the L2 stage that is abolished by adulthood. Note that the DD ratio at the L2 versus A2 ages is similar (B), whereas the DD ratio at L3 is higher than at A1 (E). ns: not significant, *P < 0.05, **P < 0.01, ***P < 0.001, ****P < 0.0001, two-way ANOVA with uncorrected Fisher's LSD, with single pooled variance.

*C. elegans* has a series of GABAergic motor neurons with cell bodies along the ventral nerve cord. A subset of these, called the VD neurons, makes presynapses along the ventral nerve cord. In the rest, called the DD neurons, presynapses are located along the dorsal nerve cord for most of the animal's life. Quantifying SNG-1::ARGO GFP/RFP in the VD and DD neurons at different animal ages, we found a strong age-by-neuron-identity interaction in regulating steady-state presynaptic SNG-1::ARGO GFP/RFP (Figure 5). At the L2 larval stage, the VD neurons’ mean ratio was 2.5, whereas the DD neurons’ mean ratio was less than 1.5 (Figure 5, A and B). Presynapse size, based on the SNG-1::ARGO RFP intensity, was similar in L2 larvae between the VD and DD neurons (Figure 5C). The DD neurons’ GFP intensity was low in L2 larvae, though (Figure 5D). We speculate that this difference between VD and DD in L2 larvae is related to the distinct mechanisms of synaptic development between these two otherwise similar classes of neurons. In the L1 larval stage, the DD neurons’ presynapses are located along the ventral nerve cord, and the VD neurons have not yet been generated. At the end of the L1 stage, the DD neurons’ presynapses are relocated to the dorsal nerve cord (White *et al.*, 1978; Hallam and Jin, 1998). This remodeling involves moving the pre-existing presynaptic proteins from the ventral to the dorsal side (Park *et al.*, 2011). Meanwhile, the VD neurons are born, and they generate their initial presynapses along the ventral nerve cord, where they remain. By imaging the VD/DD neurons in the L2 stage, we visualized the synapses shortly after this DD remodeling and VD synaptogenesis event. To fill in the age gap between L2 and A2, we performed a separate experiment in which we assessed steady-state SNG-1::ARGO in the VD versus DD neurons at L3 and A1 (Figure 5, E–G). In both neuron classes, the SNG-1::ARGO GFP/RFP ratio is significantly higher at L3 than at A1 (Figure 5E). Considering these results together with the L2 versus A2 experiment suggests that the especially low SNG-1::ARGO ratio in the DD neurons during or directly after presynapse remodeling is a transient condition, and the ratio increases by the next larval stage.

Based on these results, we speculate that when SNG-1::ARGO in the DD neurons is relocated from the ventral to the dorsal nerve cord, it is trafficked within acidified endosomes, wherein the GFP is quenched. Both the SV-transporting kinesin KIF1A/UNC-104 and the more generalist Kinesin-1/UNC-116 are required for DD presynapse remodeling; consistent with our speculation, UNC-116 can transport endosomal and autophagic compartments (Klassen *et al.*, 2010; Park *et al.*, 2011; Kurup *et al.*, 2015; Stavoe *et al.*, 2016). In this speculation, the SNG-1::ARGO would be sorted out of the acidified compartments and into SVs in the newly formed DD dorsal presynapses.

### Additional assessment of the ARGO method using UNC-11/AP180::ARGO

**FIGURE 6:**
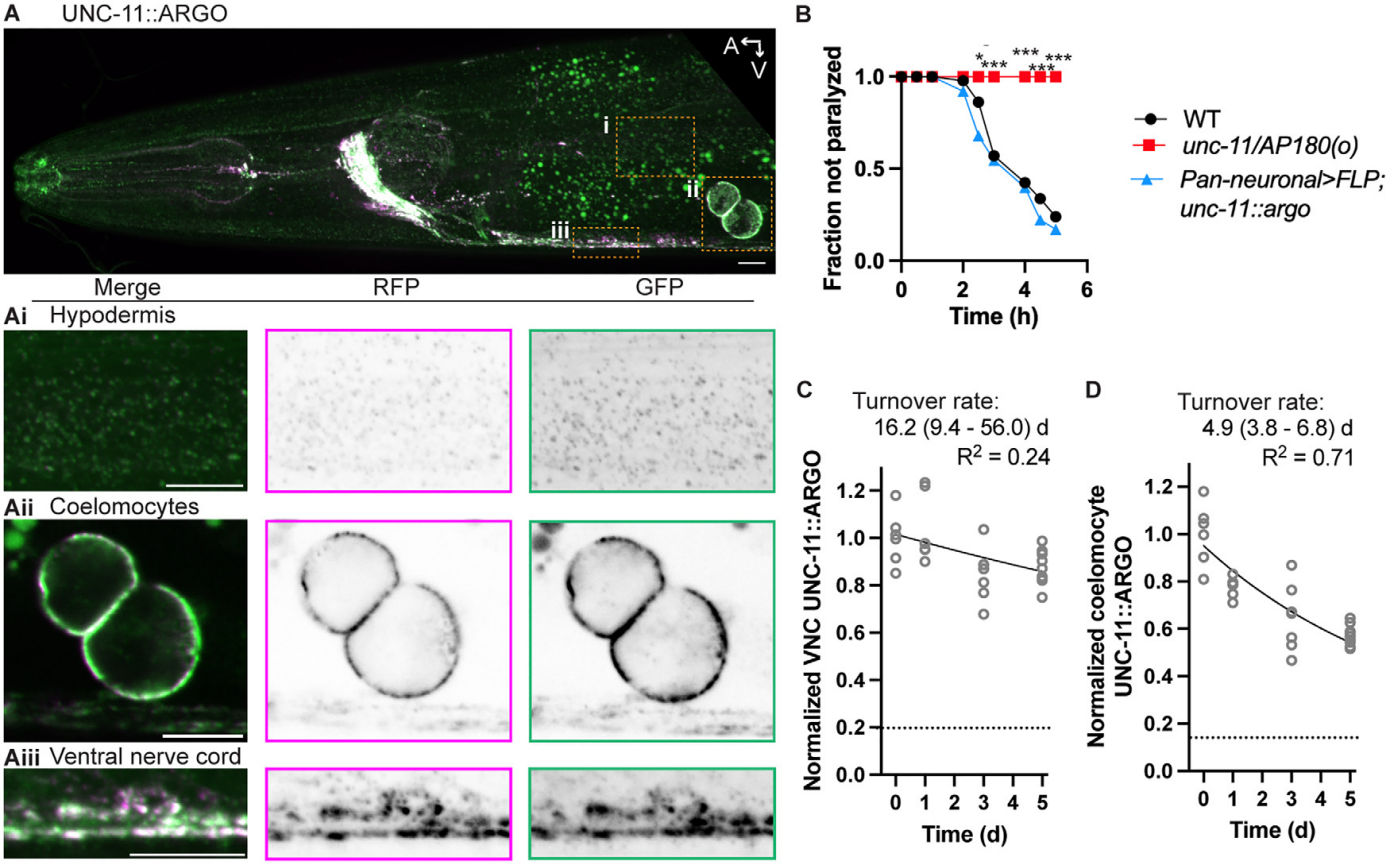
ARGO-tagged clathrin adaptor UNC-11/AP180 exhibits wild-type function and an apparent turnover rate that varies by cell type. (A) Representative image of the head of an animal with the *unc-11::argo* allele in which the ARGO tag is flipped on ubiquitously, likely due to leaky expression of the pan-neuronally expressed FLP. Zoomed-in views below show (Ai) the hypodermis (1.2-µm thick z-section), (Aii) two coelomocytes (0.4-µm thick z-section), and (Aiii) the ventral nerve cord (VNC), including one neuron soma (2.4-µm thick z-section); scale bar, 10 µm. (B) Sensitivity to 0.75 mM aldicarb in day 1 adults (A1) shows that the UNC-11::ARGO does not notably impact neurotransmission, in contrast with the null allele *unc-11(e47)* (*P < 0.05, ***P < 0.001, Fisher's exact test with Bonferroni correction, *n* = 90 animals per genotype summed across three separate experiments). (C and D) UNC-11::ARGO turnover experiment with the pulse at A2. A one-phase exponential decay function was fit to the data; each data point shows the average UNC-11::ARGO GFP/RFP ratio from all the puncta in the posterior VNC (C) or a coelomocyte (D), normalized to the mean ratios from age-matched, nonpulsed controls. The dashed black line shows the experimentally calculated background, which comes from autofluorescence. (Half-life quantification is the best-fit value and 95% CI).

To further validate the ARGO method, we made an *argo* allele for UNC-11/AP180, an endocytic adaptor. In neurons, UNC-11 prominently localizes to presynapses, and it promotes SV endocytosis during SV cycling (Nonet *et al.*, 1999). An endogenous *unc-11::gfp* fusion has been made previously, which did not detectably disrupt *unc-11* function; UNC-11::GFP was detected in a variety of tissues beyond neurons, including coelomocytes and hypodermis (Gallegos *et al.*, 2021). Likewise, the ubiquitously flipped-on UNC-11::ARGO is present at presynapses, around the cortex of coelomocytes, and at numerous small puncta, likely endocytic pits, in the hypodermis (Figure 6A). Whereas the *unc-11(e47[null[)* mutant is small and uncoordinated compared with wild-type, the pan-neuronally flipped-on *unc-11::argo* animals appear to behave like wild-type (Supplemental Videos S1–S3). Furthermore, whereas the *unc-11(e47[null])* mutant is strongly resistant to aldicarb, the pan-neuronally flipped-on *unc-11::argo* animals exhibit aldicarb sensitivity indistinguishable from wild-type (Figure 6B). These data indicate that the ARGO tag does not inherently disrupt the tagged protein's function. Finally, we performed turnover UNC-11::ARGO turnover experiments in a strain with ubiquitously flipped-on *unc-11::argo,* with the pulse at A2 of adulthood. Along the ventral nerve cord (VNC), the apparent turnover rate was 16.2 d (95% CI, 9.4 – 56.0 d) (Figure 6C). In the coelomocytes, by contrast, the UNC-11::ARGO turnover was more than three times faster, with an apparent turnover rate of 4.9 d (95 CI, 3.8–6.8 d) (Figure 6D). This wide range in turnover kinetics, combined with the aldicarb data, further supports the notion that ARGO reports the tagged protein's turnover rate without impacting it.

## DISCUSSION

In summary, we developed and validated ARGO, a genetically encoded method to easily and inexpensively quantify protein turnover in vivo with high spatial and temporal resolution, and we used this method to begin to assess how transmembrane SV protein Synaptogyrin/SNG-1 is turned over from the presynapses in adulthood. These findings provide new insights into SV protein degradation, and they illustrate the utility of the ARGO method.

### The ARGO method

The abundance of each of a cell's proteins is determined by its rates of production and degradation, plus the rate of cell division (Liu *et al.*, 2016). In terminally differentiated cells such as neurons, the protein degradation rate takes a more prominent role in controlling protein abundance (Liu *et al.*, 2016). Evidence from proteomic studies indicates that protein half-life can range from minutes to months (and, rarely, even longer), depending on the protein, the cell type, and the cellular context (Toyama *et al.*, 2013). An impediment to understanding the regulation of protein degradation is the limits of existing methods for easily assessing protein degradation in vivo with high resolution. The most straightforward existing approach for interrogating a protein-of-interest's turnover is adding a self-labeling tag to that protein, most commonly SNAP-tag or HaloTag, providing a pulse of fluorescent ligand, then imaging turnover with fluorescence microscopy during the chase (Bojkowska *et al.*, 2011; Bulovaite *et al.*, 2022). ARGO is a conceptually and methodologically similar strategy with several advantages: First, because ARGO is fully genetically encoded, once the strain has been generated, performing experiments is simpler and cheaper. This makes it highly amenable to thorough interrogation of surveillance and degradation mechanisms, for example, with forward and reverse genetics. Second, because the imaging is ratiometric, it provides higher resolution of protein half-life, and it is advantageous to use with genetic or circumstantial manipulations that alter the steady-state localization of the protein-of-interest. Third, because the GFP fluorescence is sensitive to acidic environments, analysis of steady-state fluorescence can determine whether and where degradation is mediated via sorting into a lysosomal pathway. That third advantage of ARGO compared with self-labeling tags is also an advantage compared with photoconvertible fluorophores. Steady-state imaging of dually tagged fluorescent reporters of autophagy and mitophagy has been used extensively and to great effect for understanding how the location and rate of autophagy are regulated (Rudinskiy *et al.*, 2024); steady-state imaging of an ARGO-tagged protein-of-interest combined with genetic and/or environmental perturbations can be used similarly. The design of ARGO is modular, making it amenable to modification. For instance, though we used a heat shock promoter to drive Cre expression here, any temporally inducible transcription system can, in theory, be used to provide the pulse.

A distinction between ARGO, as well as the similar approaches described above, and the more classical pulse–chase approaches is the population of proteins being tracked. In more traditional pulse–chase experiments, the pulse labels a cohort of newly synthesized proteins during a brief time window, and the stability of that cohort is followed during the chase (e.g., Schubert *et al.*, 2000; Dieterich *et al.*, 2006). This type of approach is well-suited for detecting subpopulations of proteins that are rapidly degraded during or shortly after synthesis via quality control pathways, such as defective ribosomal products (Schubert *et al.*, 2000). In contrast, the ARGO method labels the entire pre-existing pool of a protein before the pulse. It is more useful for determining the fates of proteins that were successfully produced and delivered to the subcellular location in which they function. These approaches, therefore, provide complementary views of protein turnover.

### SV protein degradation

**FIGURE 7:**
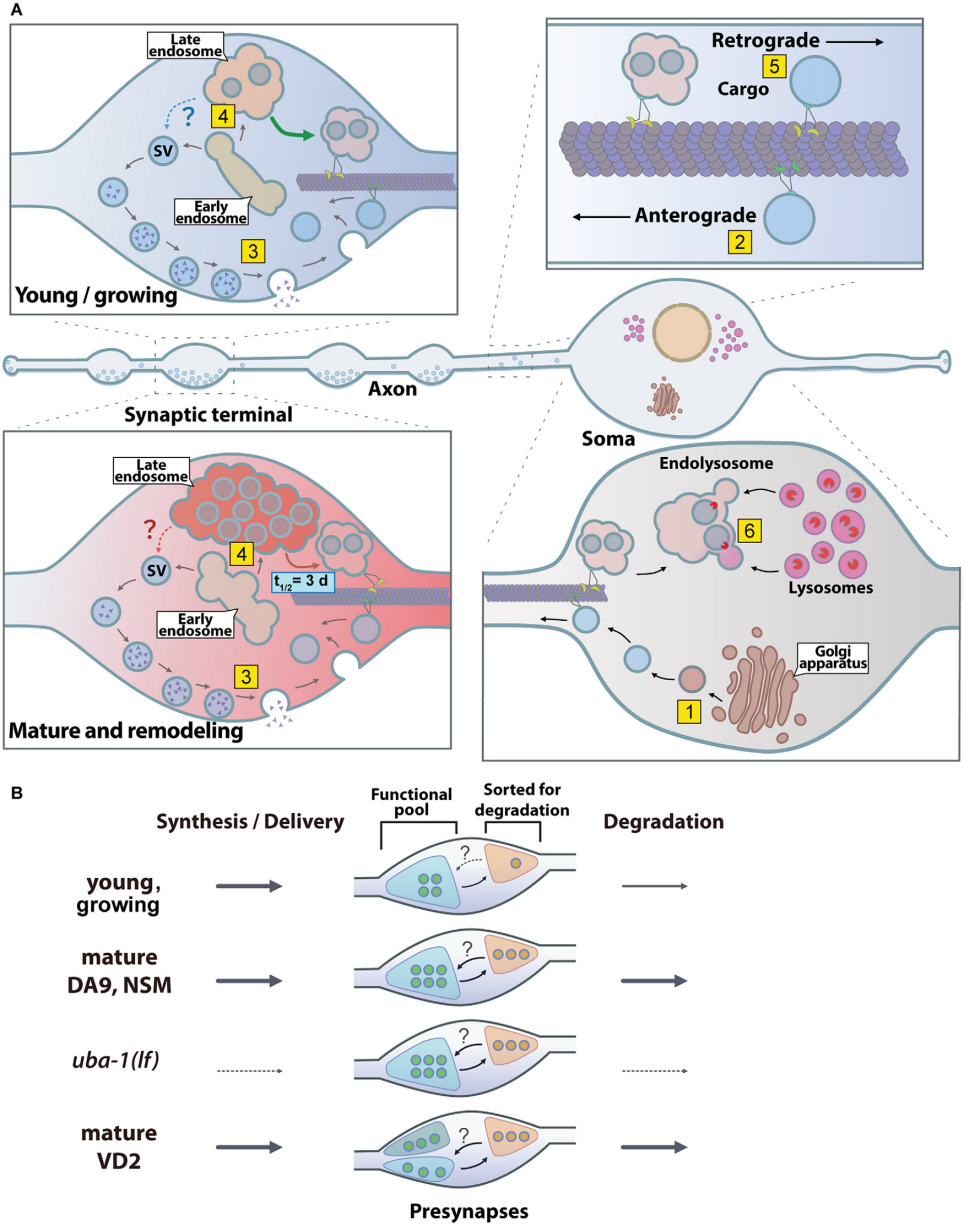
Model for SNG-1 turnover at the synapse. (A) SNG-1 turnover involves 1) synthesis in the cell body, 2) anterograde trafficking to the presynapses, 3) a duration of function in SV cycling, 4) sorting for degradation into acidic compartments at the presynapse, 5) retrograde trafficking, and 6) degradation within lysosomal compartments in the soma. SV proteins are continuously trafficked between synapses and throughout the axon as well (not depicted). In the mature neuron, a fraction of presynaptic SNG-1 resides within acidic endosomal compartments, suggestive that the removal of these sorted-for-degradation molecules from the presynapse via retrograde transport is rate-limiting for degradation. (B) In a young, growing neuron, the rate of SNG-1 removal and degradation from the presynapse (*t*_1/2_ > 3.4 d for the DA9 neuron) is exceeded by the rate of production and delivery of new SNG-1 molecules, leading to net synapse growth. In a mature neuron, the rate of SNG-1 removal and degradation from the presynapse (*t*_1/2_ = 3 d for the DA9 neuron) matches the rate of production and delivery of new SNG-1 molecules, leading to net maintenance of SNG-1 abundance. Presynaptic SNG-1 turnover—both delivery and removal—is dependent on UBA-1/E1. In one neuron type, the GABAergic VD2, the biphasic SNG-1 turnover suggests two nonintermixing pools of SNG-1 within each presynapse.

Our steady-state imaging data indicate that SNG-1 is sorted for degradation into acidic endosomal compartments at the synapse, then transported to the cell body for degradation in lysosomal compartments (Figure 7A). This corroborates the most prevailing, though sometimes disputed, model for the spatial dynamics of SV protein degradation (Jin *et al.*, 2018; Winckler *et al.*, 2018). This model is further supported by recent work showing that Transcription Factor EB (TFEB)/HLH-30 promotes expansion of neuronal lysosomal capacity in early adulthood and, in the absence of *hlh-30*, presynaptic SNG-1 turnover is slowed compared with wild-type by ∼50% (Zhong and Richardson, 2025).

We found that the rate of SNG-1 turnover is similar across all the *en passant* presynapses within a neuron, regardless of their position relative to the cell body (Figure 3C; Supplemental Figure S5J). This contrasts with postsynaptic protein PSD95, for which the turnover rate differs between postsynapses within a single neuron based on their spatial positioning in the mouse brain (Bulovaite *et al.*, 2022). Perhaps the continuous trafficking of SVs between synapses generates the conditions in which SNG-1 functions as a single super-pool in regard to turnover (Staras *et al.*, 2010; Herzog *et al.*, 2011; Wu *et al.*, 2013). Alternatively, the molecules and mechanisms that regulate the rate of SNG-1 degradation could be effectively uniform across presynapses.

The steady-state presynaptic SNG-1::ARGO GFP/RFP ratio varies with both neuron identity and age. We interpret this variation primarily as reflecting differences in the proportion of SNG-1::ARGO residing in acidic compartments because 1) sorting into acidic compartments is the canonical route for transmembrane protein degradation, 2) the ratio increases upon injection with Bafilomycin A (Figure 2E) and concanamycin A (Zhong and Richardson, 2025), and 3) the marked difference in presynaptic ratio between VD and DD neurons in L2 larvae is not readily explained with alternate, technical interpretations (Figure 5). Still, we cannot exclude that some factor might contribute to changes in SNG-1::ARGO ratio, such as misfolding of GFP on a subset of newly synthesized SNG-1::ARGO molecules. Importantly, the quantification of SNG-1::ARGO half-life normalizes against the GFP/RFP ratio in age-matched control animals and is therefore unaffected by the variation in steady-state ratio.

Positing that the SNG-1::ARGO GFP/RFP ratio mainly reflects sorting into acidic compartments, our data indicate that a large proportion of presynaptic SNG-1 resides in acidic compartments in adult neurons and in newly remodeled DD synapses. These acidic compartments are likely late endosomes and/or autophagosomes. Have these SNG-1 molecules been decommissioned and committed to degradation? This notion seems surprising, yet it aligns with findings from cultured hippocampal neurons showing that SV proteins participate in SV cycling for less than half of their lifetime and, once targeted for degradation, often continue to reside at the presynapse (Truckenbrodt *et al.*, 2018). On the other hand, sorting into acidic endosomal compartments need not entail an irreversible exit from the SV cycle: intraluminal vesicles within late endosomes can undergo back-fusion, allowing resident proteins to rejoin the functional pool (Eden and Futter, 2021; Perrin *et al.*, 2021). It is also possible that SNG-1 has an unappreciated function within endosomal compartments. For the DD synapses in L2 larvae in particular, it would be puzzling if SNG-1 were relocated from the original ventral presynapses to the newly generated dorsal presynapses only to be rapidly eliminated.

Our results indicate that reduction-of-function of the sole *C. elegans* E1 ubiquitin ligase, *uba-1*, dramatically slows SNG-1 turnover from the presynapse (Figure 3J). Interestingly, the slowed presynaptic SNG-1 turnover in the *uba-1(it129ts)* mutant is not accompanied by an observable increase in SNG-1::ARGO accumulation anywhere in the neuron (Supplemental Figure S7). Two phenotypes together suggest that the rate of SNG-1 production is adjusted based on the rate of degradation or vice versa. We also show that blocking proteasomal degradation with bortezomib decreases SNG-1::ARGO turnover without causing presynaptic SNG-1::ARGO buildup; this result may involve some overlap in mechanism as the *uba-1(it129ts)* result. As UBA-1 and the proteasome broadly promote protein degradation, one model to explain these data would be that severely depressed protein degradation leads to a lack of amino acids from which to build new proteins (Suraweera *et al.*, 2012). In that model, the primary defect with both *uba-1(it129ts)* and bortezomib would be a lack of SNG-1 synthesis. The secondary effect would be slowed SNG-1 degradation from the synapse, which would invoke an ability to adjust the rate of SNG-1 degradation in order to maintain the appropriate abundance. A second model is that when proteasomal degradation is defective, autophagy is homeostatically increased to help maintain the proteome, but lysosomal capacity is not sufficiently expanded, leading to a backup in lysosomal degradation (Dikic, 2017). A third possibility is that removal of SNG-1 from the synapse for degradation requires the ubiquitin+proteasome–mediated degradation of some other specific protein(s) whose function is to negatively regulate SNG-1 turnover. For both the second and third models, the posited primary defect in SNG-1 turnover is in SNG-1 degradation, necessitating some secondary mechanism to prevent the excess production and/or accumulation of SNG-1. That secondary mechanism may involve homeostatic feedback that reduces protein synthesis and/or exocytosis of excess SNG-1 protein from the cell, for instance, in exosomes. As a final consideration, the primary defect mechanism from multiple of these models might be occurring simultaneously, obviating the need for a feedback mechanism.

Finally, our data suggest that in at least one *C. elegans* neuron type—the GABAergic motor neuron VD2—SNG-1 exists in two distinct pools within each presynapse that do not intermix over at least several days. This is surprising because SV cycling is generally thought to intermix vesicle proteins in an endosomal sorting compartment, effectively generating a single pool of SV protein over long timescales. We consider several ways in which two SNG-1 half-lives could arise. One possibility is that SNG-1 resides on two distinct SV pools in the VD2 neuron. To remain chronically distinct, one pool might never participate in neurotransmitter release, the pools might operate in temporally or spatially distinct SV cycling, or one or both pools might exclusively use kiss-and-run fusion, bypassing endosomal sorting. Hints from other experimental animals suggest these scenarios might be plausible. For instance, a synapse can contain a “resting” SV pool that is resistant to recruitment upon strong experimental stimulation, might not cycle under some physiological contexts, and can comprise over half of the SVs in a presynapse (Chanaday *et al.*, 2019). SVs that do release can be further separated into the evoked release versus the spontaneously released pools, and in some contexts, these pools remain segregated for tens of minutes (Sara *et al.*, 2005; Mathew *et al.*, 2008; Andreae *et al.*, 2012; Kavalali, 2014; Egashira *et al.*, 2022). Nonetheless, we are not aware of examples where SV pools retain their distinct identities for days. Another possible explanation is that SNG-1 could itself exist in two distinct forms, such as differential posttranslational modification(s) during SNG-1 synthesis or a developmental time window, with one form resistant to degradation. Various mammalian synaptogyrin isoforms can carry posttranslational modifications, though none have been identified for *C. elegans* SNG-1 (iPTMnet) (Huang *et al.*, 2018). Further research on this two-phase SNG-1 turnover will illuminate how SV proteins can achieve selective turnover, which could have broad implications for how neurotransmission is tuned.

## MATERIALS AND METHODS

Request a protocol through *Bio-protocol*

### *C. elegans* culture and maintenance

Nematodes were grown at room temperature on nematode growth media (NGM) plates seeded with *Escherichia coli* OP50. All experiments used hermaphrodite worms and the stages used for each experiment are outlined in each figure legend. Several strains were provided by the *Caenorhabditis* Genetics Center (CGC), which is funded by the NIH Office of Research Infrastructure Program (P40 OD010440). The *carIs1(Pglr-4 > Flippase)* allele, which flips on *sng-1::argo* in the DA9 neuron, was generated by integrating *shyEx246* from the Shaul Yogev laboratory. To induce expression of *heSi160[Pheat-shock > Cre]* for the ARGO pulse, worm plates were shifted to a 34°C incubator for 1 h. For experiments with *uba-1(it129ts)*, animals were grown at 20°C, then shifted to 25°C, the nonpermissive temperature, at the L2 larval stage. Consistent with reported phenotypes, these animals at 25°C were fully sterile. The canonical permissive temperature for *uba-1(it129ts)* is 15°C, where the allele behaves like a weaker hypomorph; in our hands, the strain is viable and appears grossly wild-type at 20°C. Supplemental Table S1 lists the strain information and setup for all experiments. Supplemental Table S2 lists strains used in this study.

### Cloning and transgenic strain generation

Cloning was performed using the pSM vector, a derivative of pPD49.26, using standard restriction enzyme and Gibson cloning techniques. The *Podr-1>gfp* was provided by the Kang Shen lab, Stanford University. To make pSPW005(Pmig13>nuc-1::tagBFP2), *nuc-1* cDNA and *mTagBFP2* were subcloned from pRZ1 and pMA024, respectively, while the backbone, which included the *Pmig-13* promoter, was cloned from pCER286. pMA028(Pmig-13>sng-1::dendra2) was cloned using Gibson assembly; *dendra2* was amplified from pMDJ17, and it was combined with *Pmig-13>sng-1* from pCER286. Extrachromosomal transgenes were generated with standard microinjection methods. To generate *carSi11[loxP Pmig-13>sng-1::Dendra2 FTR3] IV,* the *Pmig-13>sng-1::Dendra2* construct from pMA028 was cloned into the pHygRR2 vector (Addgene plasmid #200075) and injected into the *jsSi1669 IV; sng-1(ok234) X* landing pad strain. We performed hygromycin selection and subsequent selection marker removal as previously described (Nonet, 2023).

### Genome editing using CRISPR/Cas9

The initial *argo* allele, *sng-1(syb3140)*, consisting of *FRT-stop+3′utr-FRT-RFP-loxP-GFP-loxP,* was generated by SunyBiotech. We determined that the resulting SNG-1::RFP::GFP was destabilized and had accelerated degradation. Removal of GFP upon ARGO activation led to increased RFP fluorescence intensity up to the expected intensity based on existing strains (Schwartz and Jorgensen, 2016), indicating that the amino acid linker between the RFP and GFP was causing the destabilization. We therefore used CRISPR/Cas9 to modify the amino acid linker between the RFP and GFP in the *sng-1(syb3140)* allele, generating the *sng-1(syb3140car2)* allele, which fixed the destabilization. The *syb3140car2* allele was used in all experiments in this study with two exceptions: Supplemental Figure S1M, which quantifies the rate of Cre-mediated recombination, and Table 1, which quantifies Cre/loxP specificity and efficacy. Both of these parameters are expected to be identical between *syb3140* and *syb3140car2*. The *car2* allele was generated following the protocol in Paix *et al.* (2015). A ssDNA with 36 and 38 bp homology on either side was used for the repair template (Ultramer, Integrated DNA Technologies [IDT]). Two cRNAs (IDT) were used, one on either end of the edit. The genomic sequence of the *argo* cassette and the cDNA sequences of *sng-1::argo* before and after the removal of *GFP* are provided in Supplemental Figures S4–S6.

### Microscopy and imaging

Worms were mounted on 3% freshly made agarose pads into drops of 10 mM levamisole. An imaging system composed of a Nikon Eclipse Ti2 microscope, Yokogawa CSU-W1 SoRa spinning-disk unit, and a Hamamatsu ORCA-fusionBT digital camera C15440 with Plan Apo x60 A1/2 Nikon objective with water immersion was used to acquire all images. Image settings (60% of laser power and 200 ms exposure time) were identical for all groups and treatments across experiments. Z-stacks were taken with a 0.4-µm step in the 20-30 µm range, depending on the type of neuron. For super resolution by optical reassignment (Figure 1I), the 2.8x SoRa magnifier was used, z-stacks were acquired with 0.3 µm steps, and images were deconvoluted in postprocessing.

### Aldicarb assay

Aldicarb assays were performed as described (Mahoney *et al.*, 2006). Briefly, 30 day 1 adults of each strain were placed on a plate containing 0.5 mM aldicarb, and they were periodically assessed for paralysis visually after touch with an eyelash. Plates were scored blind to genotype, and the experiment was executed in three biological replicates, each on a separate day.

### qPCR of genomic DNA

Day 0/L4 animals of the genotype *bqSi542; sng-1(syb3140) heSi160* were shifted to 34°C for 1 h at Time = 0. Directly before the shift, and at the indicated times afterward, ∼50 animals were picked and lysed with the standard worm lysis protocol. Relative abundance of the Cre-recombined locus was assessed using primers that aligned to the 3′ end of *RFP* before the first *loxP* and the 3′ end of *sng-1* after the second *loxP.* Relative transcript abundance was calculated using the ΔΔ*C*_t_ method, with primers against *gpi-1* used as the internal control. All reactions were performed in triplicate.

### RNA extraction and RT-qPCR

Total RNA was extracted from *C. elegans* samples using the TRI reagent (Sigma-Aldrich), ethanol precipitation protocol. The LunaScript Kit (NEB) was used for complementary DNA (cDNA) synthesis according to the manufacturer's protocol. Quantitative RT-PCR was performed using the Roche Lightcycler 480. Primers for the target genes (*sng-1, hsp-16*) and the reference/normalization gene (*pmp-42*) were validated empirically for specificity and efficiency. Melting curve analysis was performed at the end of each run to confirm amplification specificity. Gene expression levels were calculated using the ΔΔ*C*_t_ method, with the *pmp-42* gene serving as the internal control. All reactions were performed in triplicate.

### Analysis of NUC-1::BFP and BFP::RAB-3 localization compared with SNG-1::ARGO

PBT333 *carIs1(Pglr-4>Flp, Podr-1>rfp); sng-1(syb3140car2); carEx20(Pmig-13>nuc-1::tagBFP2, Podr-1>gfp)* and PBT292 *carIs1(Pglr-4>Flp, Podr-1>rfp); sng-1(syb3140car2); carEx16(Pmig-13>tagBFP2::rab-3, Pmyo-2>gfp)* were imaged at A3. For the former, the NUC-1::BFP signal was present only in the soma; therefore, only this compartment was analyzed further. Because the GFP channel for SNG-1::ARGO was the brightest and most prominent over background signal, it was used to create a mask for particle analysis in ImageJ, and this mask was then applied to the other channels. For the latter, analysis was similar, but in addition, each channel was used to make a mask separately for Supplemental Figure S3A. Only the mean intensity fluorescence for each channel and their ratios were used for the presented plots.

### Bafilomycin A treatment

Bafilomycin A was injected into the body cavity of *C. elegans*, similarly to previous work (Wilkinson *et al.*, 2015). Briefly, 25 µM of BafA in 5% DMSO or 0.2% DMSO (“mock”) was coinjected with Orange G for color into the pseudocoelom, and animals were allowed to recover for 3 h before they were imaged with fluorescence confocal microscopy as described above. BafA-injected animals were still moving when they were put on slides, indicating that the BafA had not completely blocked V-ATPase function. We selected animals that were still able to move to ensure that they were alive. This criterion was important because death could alter cellular properties relevant to presynaptic architecture, including molecular crowding, ionic concentrations, and membrane integrity. Such disruptions would be problematic for interpreting any increase in SNG-1::ARGO GFP/RFP ratio as indicative of deacidification of lysosomal compartments.

### RNAi treatment and analysis

For RNAi treatment, L4 worms (P0) were transferred to 1 mM IPTG plates seeded with HT115 *E. coli* containing empty vectors, *unc-22* RNAi, or *sng-1* RNAi clones from the Ahringer Library (Kamath *et al.*, 2003). The feeding was continued in the progeny and throughout the experiment. L4/A0 worms were picked from the F1 generation and imaged at day 2 of adulthood. To quantify the effect of *sng-1* silencing, the axon, commissure and the cell body of DA9 neuron from each worm was scored based on the following categories: worms with apparent SNG-1::ARGO fluorescence as bright as untreated worms were counted as “bright;” worms with dimmer SNG-1::ARGO fluorescence compared with untreated worms were counted as “dim;” worms without visible SNG-1::ARGO fluorescence were counted as “not visible. Statistical analysis was performed in GraphPad Prism 10. Fisher's exact test was performed between *sng-1* RNAi and control RNAi.

### Assessing the specificity and efficacy of the pulse

To assess the efficacy and specificity of Heat-shock>Cre-mediated recombination of *GFP* from the *argo* cassette (Table 1), fluorescence was scored based on visualizing SNG-1::ARGO GFP and RFP fluorescence at least 3 d after activation. Scoring was performed using a combination of two approaches: 1) qualitative assessment by eye on a compound fluorescent microscope, wherein the experimenter performed an initial visual assessment of a subset of animals and then binned all of the animals in the cohort as either “bright” (no recombination) or “dim” (recombination); 2) semiquantitative assessment on the spinning disk confocal microscope, wherein the animals were imaged with standardized microscope settings, raw fluorescence intensity and GFP/RFP values were assessed for a subset of animals, and then all animals in the cohort were binned as “ratio high” (no recombination) or “ratio low”(recombination). The distribution of GFP/RFP ratios in all instances was bimodal; if an animal appeared to have an intermediate value, which occurred in about 1-5% of individuals, that animal was not scored.

### Microscopy data analysis of SNG-1::ARGO at synapses

Nd2 files were screened with NIS Elements Software for unusually low signal (indicative of hardware malfunction or a mistake in imaging setup), severe movement or drifting, and unusable images were discarded. The remaining images were processed with FIJI (ImageJ) software. Before quantification, each image was assessed for animal position, and only animals in the optimal position were included. “Optimal position” is defined as laterally oriented animals (i.e., not rolled with the ventral or dorsal side facing the coverslip) with the neuronal region of interest (ROI) positioned at or above the midline of the animal relative to the coverslip. Image stacks were transformed using the z-projection function with max intensity option, areas of interest were selected either with the Rectangle tool (for cell bodies) or the Segmented line and Straighten function (for synaptic areas). For each image, a Threshold was applied with default settings to the RFP channel, and the Analyze Particles function was used to identify ROIs in that thresholded image. These ROIs were then used to quantify fluorescence from both the RFP and GFP channels. The mean intensity of each channel was used for all calculations. Each obtained array of values in CSV format was united in a single data frame, and synaptic puncta less than 0.1 µm² (for the wild-type DA9 and NSM datasets) or 0.2 µm² (for the VD, DD, and *uba-1* mutant datasets) were filtered out due to high variability in relative RFP:GFP fluorescence intensities. This high variability was likely due to low signal-to-noise; a major contributing factor to the noise was autofluorescence. The GFP/RFP ratio calculation was done for each presynapse with Jupyter Notebook and Python scripts.

To find the average presynaptic GFP/RFP ratio per neuron, all the individual GFP/RFP ratios within the presynaptic region of a neuron were averaged.

To find the average presynaptic GFP/RFP ratio by position along the proximal–distal axis, the length of each neuron's presynaptic region was divided into the most proximal 30%, the most distal 30%, and the middle 40%, and all the presynaptic puncta within each domain were averaged.

We note that the GFP/RFP ratio is sensitive to precise microscope adjustments and varied slightly between experiments performed months apart, despite our efforts to maintain consistent imaging conditions. Therefore, the SNG-1::ARGO GFP/RFP ratio should not be compared across experiments presented on separate graphs.

### Photoconversion with Dendra2

Approximately 120 animals were washed to remove residual OP50 bacteria. Animals were picked from seeded plates onto a 50 µl drop of M9 on a decontaminated plate lid and pipetted up and down to disperse adherent bacteria. This was followed by a 50 µl addition of M9 buffer to the existing 50 µl droplet with worms. Subsequently, 50 µl of supernatant was carefully removed while avoiding aspiration of animals. This washing procedure was repeated up to three times until the droplet appeared visually clear, minimizing turbidity that could interfere with photoconversion. The remaining 50 µl droplet was supplemented with 5 µl of 20 mM levamisole and incubated for 5 min. Animals should only be partially immobilized to prevent intestinal bursting from the vulva that reduces the yield of photoconverted worms.

Photoconversion was performed using an ECHO Revolve R4 fluorescence microscope equipped with a 4x objective. LED Illumination was applied through the DAPI channel (excitation 385/30 nm, emission 450/50 nm, dichroic 425 nm) at 100% intensity, configured for top illumination to ensure direct excitation of the sample. The droplet was positioned within the illuminated field and exposed to excitation light in 30-s intervals, followed by a 30-s rest period. This cycle was repeated for a total of 10 intervals.

Following photoconversion, animals in the M9 droplet were diluted with 100 µl of M9 and transferred back to seeded NGM plates by gentle pipetting. Animals adhering to the inner walls of the pipette tip were promptly released by rinsing with additional M9 using three prealiquoted 30 µl droplets on the postphotoconversion plate. This process was repeated until all animals were recovered.

Animals were imaged immediately after absorption of M9 into the plate or aged at 20°C and imaged at subsequent time points. Image settings were broadly the same as those for ARGO, except that for the red channel, the exposure was increased to 350 ms in order to detect an adequate signal. In addition, for the experiment shown in Supplemental Figure S6C, a broad bandpass emission filter was used for the red channel to collect more signal.

### Bortezomib treatment

The DA9 SNG-1::ARGO strain (*carIs1; sng-1(syb3140car2); heSi160*) was grown to A0/L4 on regular NGM plates and then transferred to regular NGM plates versus NGM plates containing 8 µM of bortezomib (Alexander *et al.*, 2023). The bortezomib was first prepared as a 5 mM stock solution in 70% ethanol, which was stored at –20°C, protected from the light. The bortezomib stock solution was diluted in water, then 200 µl of that diluted solution was added to prepoured 6 cm NGM plates to get to the 8 µM final concentration one day before adding worms. The strain VK1243 *vkEx1243(Pnhx-2>ubiquitin-V::mCherry, Pmyo-2>gfp)* was treated in parallel as a positive control to confirm that the bortezomib worked as expected. At A2 of adulthood, half of the DA9 ARGO worms from each treatment group were heat shock-pulsed, and all animals were imaged at A5 to quantify SNG-1::ARGO fluorescence. The experiment was performed in two independent biological replicates.

### Statistical analyses

Prism GraphPad software was used to generate all plots and perform most statistical analyses. The rest of the statistical analyses were performed in Rstudio as noted below.

### Assessing the effective timing of the pulse from microscopy data

To test whether the SNG-1::ARGO GFP/RFP decreased significantly by 4 h after the heat-shock (Supplemental Figure S1, F–L), we used a linear mixed-effects model with treatment (no heat-shock vs. 4 h post heat-shock) as a fixed effect and worm as a random effect to include the GFP/RFP ratio measurements for each presynapse and account for multiple measurements per animal (lmer function, lme4 package). Residual diagnostics were assessed using the DHARMa package. For DA9 D2 and D6 (Supplemental Figure S1, H and J), data were log transformed to meet model assumptions, and the back-transformed mean GFP/RFP per animal are plotted. For DA9 D2 with *heSi160* (Supplemental Figure S1H), residual plots with the log transform still showed a deviation from normality (KS test: ns, Dispersion test: ns, outlier test, *p* = 0.012), but model assumptions were considered adequately met given the large sample size and consistency of results across multiple statistical approaches, including nonparametric tests. For NSM D2 (Supplemental Figure S1K), data were square-root transformed to meet model assumptions, and the back-transformed mean GFP/RFP per animal are plotted. Empirically, this transformation was the closest to fitting model assumptions, though residual plots still showed some deviation from normality (KS test: ns, Dispersion test: ns, outlier test, *p* = 0.016). For VD2 D2 (Supplemental Figure S1L), data did not fit the assumptions of the linear mixed model, and an adequate transformation could not be found, so the mean SNG-1 ARGO GFP/RFP ratio per animal was analyzed using a two-sided *t* test (the data distribution was not significantly different from a normal distribution).

As another approach to assess whether and to what extent a cell sometimes ectopically recombines one of the two alleles of *sng-1::argo*, we compared the average presynaptic SNG-1::ARGO GFP/RFP mean and SD in the DA9 neuron between a strain with versus without *heSi160[Pheat-shock>Cre]* and saw no significant difference (Supplemental Figure S1A).

As a third approach to assess the specificity and efficacy of recombination, we compared the interneuronal CV in the presynaptic SNG-1::ARGO GFP/RFP between control and pulsed groups. We calculated the CV for control versus heat-shocked groups at each timepoint for each neuron class. As there is one CV value per neuron class, treatment, and timepoint, these comparisons are qualitative. If the CV is roughly constant in the nonpulsed group over time, it would suggest that there is not much ectopic recombination without the pulse treatment. Similarly, if the CV is roughly constant in the pulsed group over time, it would suggest that recombination is uniformly completed in both alleles of each animal.

### Assessing variance

To assess variability in SNG-1::ARGO GFP/RFP ratios across synapses within individual neurons (intraneuronal variance), because the data roughly followed a lognormal distribution, we used Rstudio to calculate the CV as:




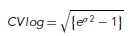




Then, the average of all CVs per worm was compared between days using the Kruskal–Wallis test, because the data were nonparametric, with Dunn's multiple comparisons test.

### Fitting decay curves to turnover data

Each SNG-1::ARGO GFP/RFP data point from the turnover experiments is the mean of all the GFP/RFP ratios from each presynaptic puncta detected in a single animal. Those data points were normalized by the mean presynaptic GFP/RFP ratio from an age-matched control (nonpulsed) cohort of animals that was imaged in parallel. In this way, the steady-state changes in presynaptic SNG-1::ARGO GFP/RFP ratio by animal age do not impact the turnover calculations. SNG-1::ARGO half-life was calculated using Prism to fit a one-phase exponential decay curve to the data. The curves were fitted using least squares regression with no weighting, considering each replicate *Y* value as an individual point. Outliers were detected and eliminated with *Q* = 5% (DA9), 1% (NSM), 0r 3% (VD2). These values were selected based on the data in Table 1, which assessed the frequency of ectopic *gfp* excision in the absence of the heat-shock pulse, and the frequency of failed *GFP* excision following the heat-shock pulse, for each neuron identity. Unless otherwise noted, we constrained the plateau to the experimentally determined value based on background GFP fluorescence, which comes from autofluorescence. This was measured from the adult day 7 images for each of the three neuron identities by hand-selecting the synaptic region in the RFP channel, copying it to the GFP channel, shifting the selection to a position adjacent to the synapses, then finding the GFP fluorescence intensities inside the ROIs. Goodness of fit was quantified with an *R*^2^.

### Assessing VD2 turnover

We noticed that for the VD2 neuron, the presynaptic SNG-1::ARGO GFP/RFP ratio does not appear to approach the background in the turnover experiment. Indeed, a two-phase exponential decay function improves the curve fit, from an *R*^2^ of 0.41 to 0.48, and comparing the one-phase versus two-phase functions using an extra sum-of-squares *F* test showed that the two-phase curve was significantly favored (Null hypothesis: one-phase decay, alternative hypothesis: two-phase decay, *P* = 0.0175). A two-phase exponential decay function will often produce a better fit than a one-phase function due to its additional parameters. Therefore, we took a second approach to assess whether the one-phase decay function could be ruled out using the same dataset: we compared the predicted plateau value from the one-phase function versus the measured background. Specifically, we fit a one-phase exponential decay function to the data without constraining the plateau. We compared the best-fit plateau and 95% CI from that function with the mean background and 95% CI calculated from the microscopy images (as described above) (Figure 4B). Indeed, the one-phase curve plateaus above the background, and the 95% CIs do not overlap (Figure 4B). Furthermore, in Figure 4D, we use the best-fit parameters from the two-phase exponential decay function from the VD2 turnover dataset in Figure 4A to predict the presynaptic GFP/RFP ratio 5 d after the pulse (the Model 1, D2 bar). That predicted value matched the real value ± SEM from a separate experiment, showing that this result is robust (the Results A2 bar).

Given that we could not find evidence for different turnover rates between individual presynaptic puncta within the VD2 neuron (Figure 4C), we considered two explanations for the two-phase turnover kinetics of SNG-1::ARGO within individual VD2 presynapses:

Model 1: There are two chronically distinct pools of SNG-1 protein
Model 2: There is a time-dependent suppression of turnover

We generated predictions from the two proposed models as described below. Then, we compared those predictions qualitatively to an experiment in which we pulsed the VD2 SNG-1::ARGO at adult D2 versus D4 of adulthood and imaged at D7 (Figure 4D). To prevent data points from inefficient or spontaneous Cre-mediated recombination from skewing the results, we censored aberrant data points as follows: the median presynaptic GFP/RFP ratio was calculated for each set of A7 animals imaged (one data point per animal, which was the mean GFP/RFP ratio across all VD2 presynaptic puncta for that animal): control, pulse at A2, pulse at A4 (all incubated at 20°C), and control versus pulse at A2 and incubated at 25°C. For the A7 controls, data points lower than the median ratio for the pulse at the A4 group were censored (3 of 51 for the 20°C controls, 0 of 20 for the 25°C controls). For all three pulsed groups, data points higher than the median ratio for the control A7 group at the corresponding temperature were censored (6 of 44, 13.6% for A2 pulse; 6 of 19 for the A4 pulse; 3 of 29 for the A2 pulse and 25°C incubation).

### I. Assessing Model I: that there are two distinct populations of SNG-1 with different turnover kinetics at each synapse

The best-fit values for the VD2 two-phase decay equation in Figure 4D are:




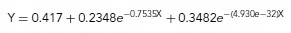




Because KSlow is essentially zero, we used the equation *Y* = 0.7652 + 0.2348e^–0.7535X^ to predict the presynaptic GFP/RFP ratio 3 and 5 d after the pulse.

### II. Assessing Model 2: that turnover is suppressed during the course of the turnover experiment

In this model, all presynaptic SNG-1::ARGO is initially subject to fast-phase turnover, but this turnover becomes inhibited or dysfunctional at a specific time in the animal's life, which occurs during the course of the turnover experiment when the pulse is at A2. In this model, only protein that undergoes fast-phase turnover before the onset of inhibition is degraded; all remaining protein is retained with unappreciable turnover thereafter. In other words, the final GFP/RFP ratio reflects the amount of SNG-1::ARGO that experienced degradation before the inhibition/dysfunction event. For the fast-phase, *k* = 0.7535 day^–1^. Solving for the time at which 60% of the protein remains gives 0.66 d after the pulse, which would equate to A2.66.

## Supporting information







Supporting Video 1 Movie S1 Mixed-stage wild-type N2 *C. elegans* behaving freely on a nematode growth media (NGM) plate show smooth, sinusoidal movements.

Supporting Video 2 Movie S2 Mixed-stage *unc-11(e47)* mutant animals exhibits uncoordinated locomotion on NGM plates. Though some animals do not move, all animals are alive.

Supporting Video 3 Movie S3 Mixed-stage *bqSi506[Pneurons>Flp]; unc-11(syb10892[argo]); heSi160[Pheat-shock>Cre]* animals appear superficially similar to wild-type during free behavior on NGM plates.

## Data Availability

Strains and plasmids are available upon request. Microscopy images will be deposited in BioImage Archive.

## Abbreviations used:

A(1, 2, 3…): adult day (1, 2, 3…)
ANOVA: analysis of variance
ARGO: analysis of red-green offset
BafA: bafilomycin A
CGC: caenorhabditis genetics center
CI: confidence interval
CV: coefficient of variation
DA9: dorsal A-type motor neuron 9
DAPI: 4′,6-diamidino-2-phenylindole
DD: dorsal D-type motor neuron
DMSO: dimethyl sulfoxide
ESCRT: endosomal sorting complexes required for transport
GABA: gamma-aminobutyric acid
GFP: green fluorescent protein
IPTG: isopropyl-beta-D-1-thiogalactopyranoside
L(1, 2, 3, 4): larval stage (1, 2, 3, 4)
NGM: nematode growth media
NP: no prediction
Ns: not statistically significant
NSM: neurosecretory motor neuron
RFP: red fluorescent protein
RNAi: RNA interference
ROI: region of interest
SEM: standard error of the mean
SV: synaptic vesicle
TFEB: transcription factor EB
Unc: uncoordinated
V-ATPase: vacuolar H^+^-ATPase
VD: ventral D-type motor neuron
VNC: ventral nerve cord.
