# Supplementary material for: Analysis in vivo using a new method, ARGO (Analysis of Red–Green Offset), reveals complexity and cell-type specificity in presynaptic turnover of synaptic vesicle protein Synaptogyrin/SNG-1
Source: Mol Biol Cell. 2026 Jun 10;37(7):ar71. doi: 10.1091/mbc.E25-09-0422 (PMC13329863; doi:10.1091/mbc.E25-09-0422)
Supplement: Supplementary file 1 [file mbc-37-ar71-s001.pdf]

Supplemental Materials

Molecular Biology of the Cell

Shiliaev *et al*.

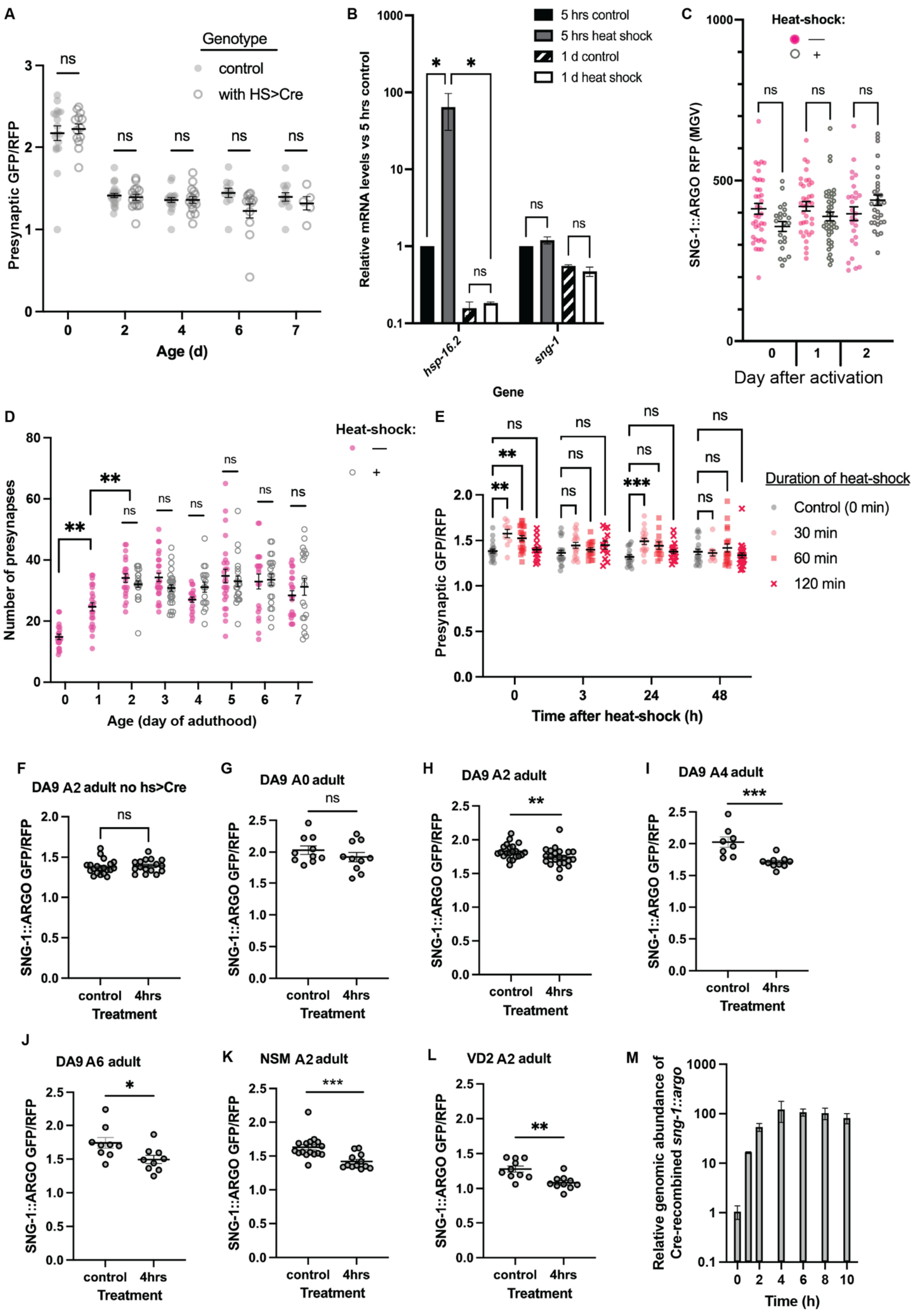
A
Genotype
control
with HS>Cre
Presynaptic GFP/RFP
Age (d)
ns
B
5 hrs control
5 hrs heat shock
1 d control
1 d heat shock
Relative mRNA levels vs 5 hrs control
hsp-16.2
sng-1
Gene
C
Heat-shock:
SNG-1::ARGO RFP (MGV)
Day after activation
D
Number of presynapses
Age (day of adulthood)
E
Duration of heat-shock
Control (0 min)
30 min
60 min
120 min
Presynaptic GFP/RFP
Time after heat-shock (h)
F
DA9 A2 adult no hs>Cre
G
DA9 A0 adult
H
DA9 A2 adult
I
DA9 A4 adult
J
DA9 A6 adult
K
NSM A2 adult
L
VD2 A2 adult
SNG-1::ARGO GFP/RFP
control
4hrs
Treatment
M
Relative genomic abundance of Cre-recombined sng-1::argo
Time (h)

Figure S1. Validation of the heat-shock-generated pulse to express Cre recombinase. (A) Steady-state average presynaptic SNG-1::ARGO GFP/RFP in a strain without versus with the *Pheat-shock>Cre* transgene *heSi160* indicates that there is little ectopic recombination in the absence of heat-shock. Each data point is the average of all individual presynaptic ratios from one DA9 neuron. (B) The heat-shock pulse does not discernably impact *sng-1* transcript levels. qRT-PCR analysis of *sng-1* mRNA levels with versus without the heat-shock conditions used as the pulse to activate SNG-1::ARGO. Values represent fold change relative to control (no heat-shock) ± SEM (n = 2 biological replicates, *P<0.05 two-way ANOVA with Tukey post-test). (C-D) The heat-shock pulse shows no significant effect on SNG-1 ARGO steady-state abundance. (C) Mean presynaptic SNG-1::ARGO RFP fluorescence intensity with versus without the heat-shock pulse. The experiment was started on A2. Each data point shows the average mean RFP intensity for all synapses of the DA9 neuron for one neuron. (D) Number of DA9 presynapses per neuron with versus without the heat shock pulse at A2. For (C-D), The heat-shocked animals at time = 0 were imaged 4 hours post heat-shock, and bars show mean ± SEM (ns: not significant, P>0.05, *P<0.5, ***P<0.001, two-way ANOVA with Tukey post-test). (E) Quantification of presynaptic SNG-1::ARGO GFP/RFP in the DA9 neuron in a strain lacking *heSi160[Pheat-shock>Cre].* Animals were heat-shocked at 34 °C for the indicated time, or not at all, at adult Day 2, then periodically imaged. Each data point is average per worm; bars show mean ± SEM (ns: not significant, P>0.5, **P<0.01, ***P<0.001, two-way ANOVA with Tukey post-test. The heat-shock used for experiments throughout the manuscript was 34 °C for one hour. (F-L) A drop in the presynaptic SNG-1::ARGO GFP/RFP can typically be detected by 4 hours post heat-shock pulse in the presence of the *Pheat-shock>Cre* transgene (G-L), but not in its absence (F). Analyses are described in Materials and Methods. The data used in F are a subset of the data from E. (M) Assessing the rate of genomic recombination to remove the *gfp* after the pulse, applied to Day 0 adults, via qPCR. Time = 0 was immediately after the 1-hour heat shock (of note, some recombination is expected to have occurred already due to the quick activation of HSF-1 to drive *Phest-shock>Cre* expression upon the initiation of heat-shock).

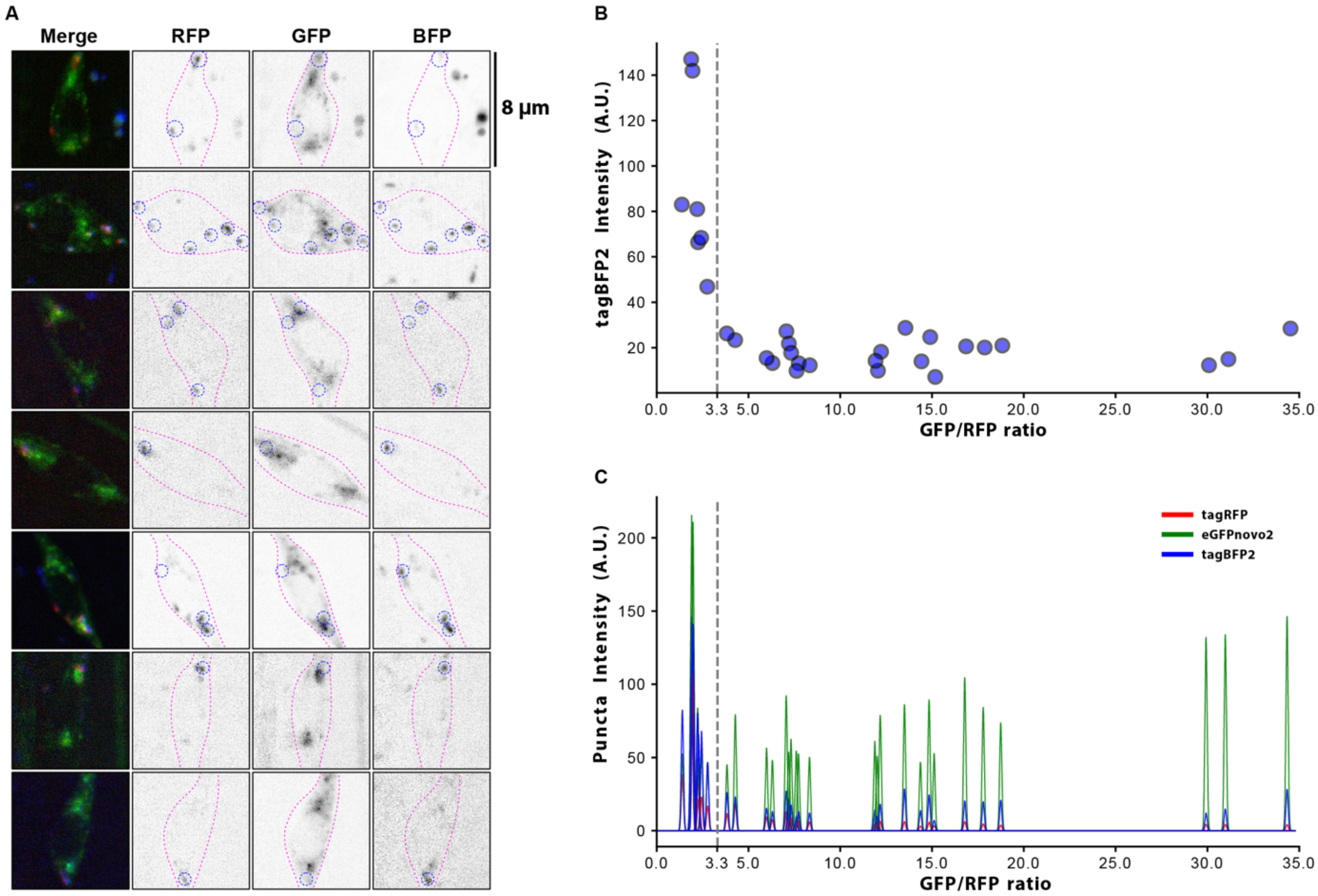

Figure S2. Lysosomal resident protein NUC-1 co-localizes with the RFP-brighter SNG-1::ARGO endosomes. (A) Seven DA9 neuron soma with flipped-on SNG-1::ARGO, co-expressing *carEx20(Pmig-13>nuc-1::tagBFP2).* Dashed magenta lines outline the neuron. Dashed blue ovals surround all NUC-1::BFP-labeled lysosomes, and the oval positions are copied to the RFP and GFP channels. (B) At each SNG-1::ARGO endosome within the soma, the fluorescence intensity of co-localized NUC-1::BFP was quantified, plotted relative to the SNG-1::ARGO GFP/RFP ratio. The dashed line indicates the theoretical ratio of this GFP variant, novo2GFP (which is brighter) to this RFP (TagRFP) when both fluorophores fluoresce at maximum intensity (plotted is 30 total SNG-1::ARGO-labeled endosomes combined from the same seven neurons shown in A). (C) An alternate plot of the same data as in B showing the intensity of RFP, GFP, and BFP in each SNG-1::ARGO endosome. This plot shows that the NUC-1::BFP intensity appears to correlate with the SNG-1::ARGO RFP intensity but not the SNG-1::ARGO GFP intensity.

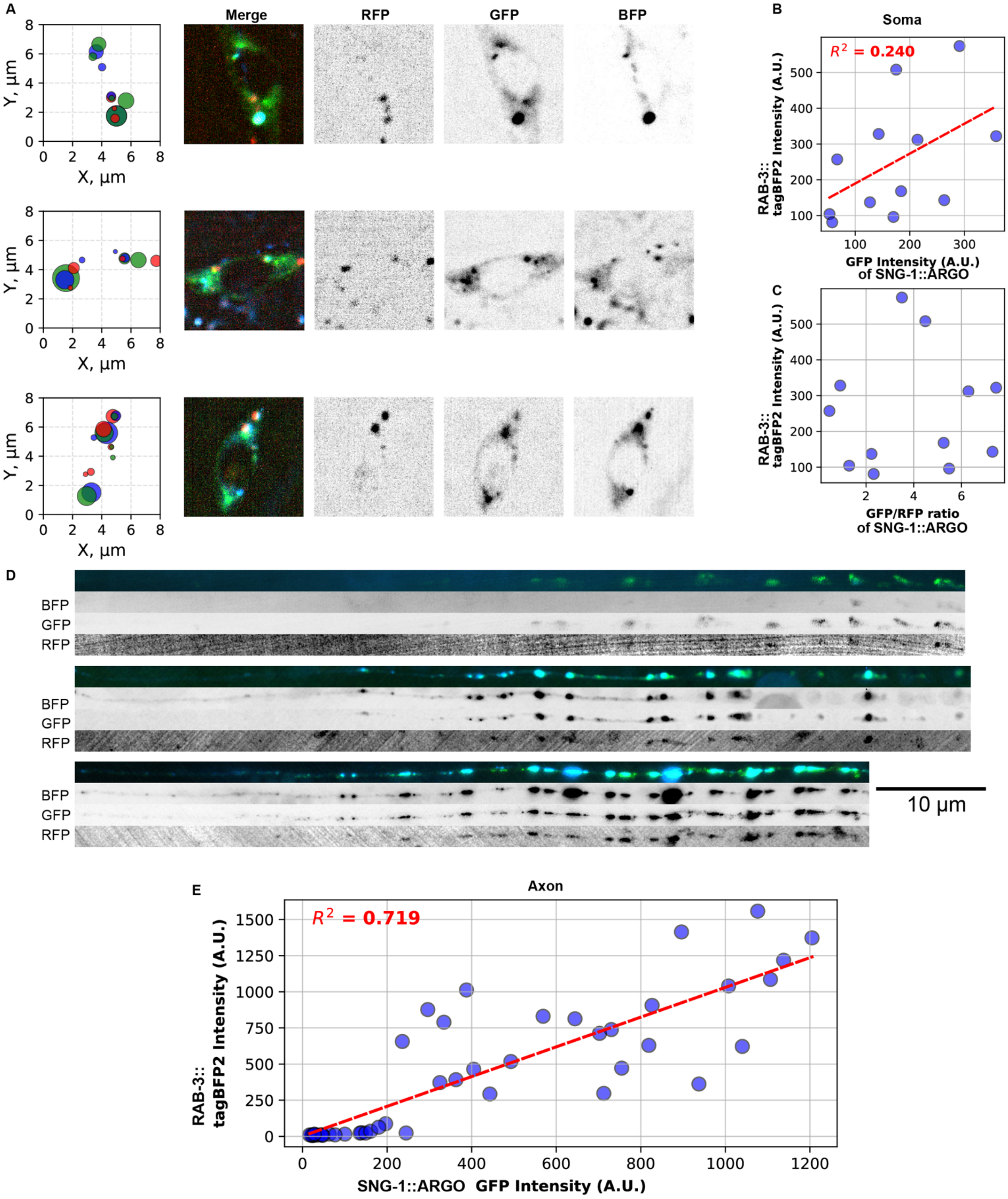

Figure S3. Synaptic vesicle protein RAB-3 co-localizes with SNG-1::ARGO regardless of the GFP/RFP ratio. (A) Three DA9 neuron soma with flipped-on SNG-1::ARGO, co-expressing *carEx20(Pmig-13>nuc-1::tagBFP2).* In the plots on the left, endosome fluorescent intensity is represented by size of each color. (B-C) The fluorescence intensity of BFP::RAB-3 at a SNG-1::ARGO endosome correlates with the brightness of

GFP (B), but not the GFP/RFP ratio (C). 12 endosomes combined from three cell bodies. (D) Line scans of the proximal portion of the DA9 presynaptic region (anterior is to the right) show that BFP::RAB-3 co-localizes with SNG-1::ARGO at presynapses, as expected. (E) Positive correlation between the intensity of BFP::RAB-3 and SNG-1::ARGO GFP at synapses. Fluorescence intensity of both is expected to positively correlate with the number of SVs at each presynapse.

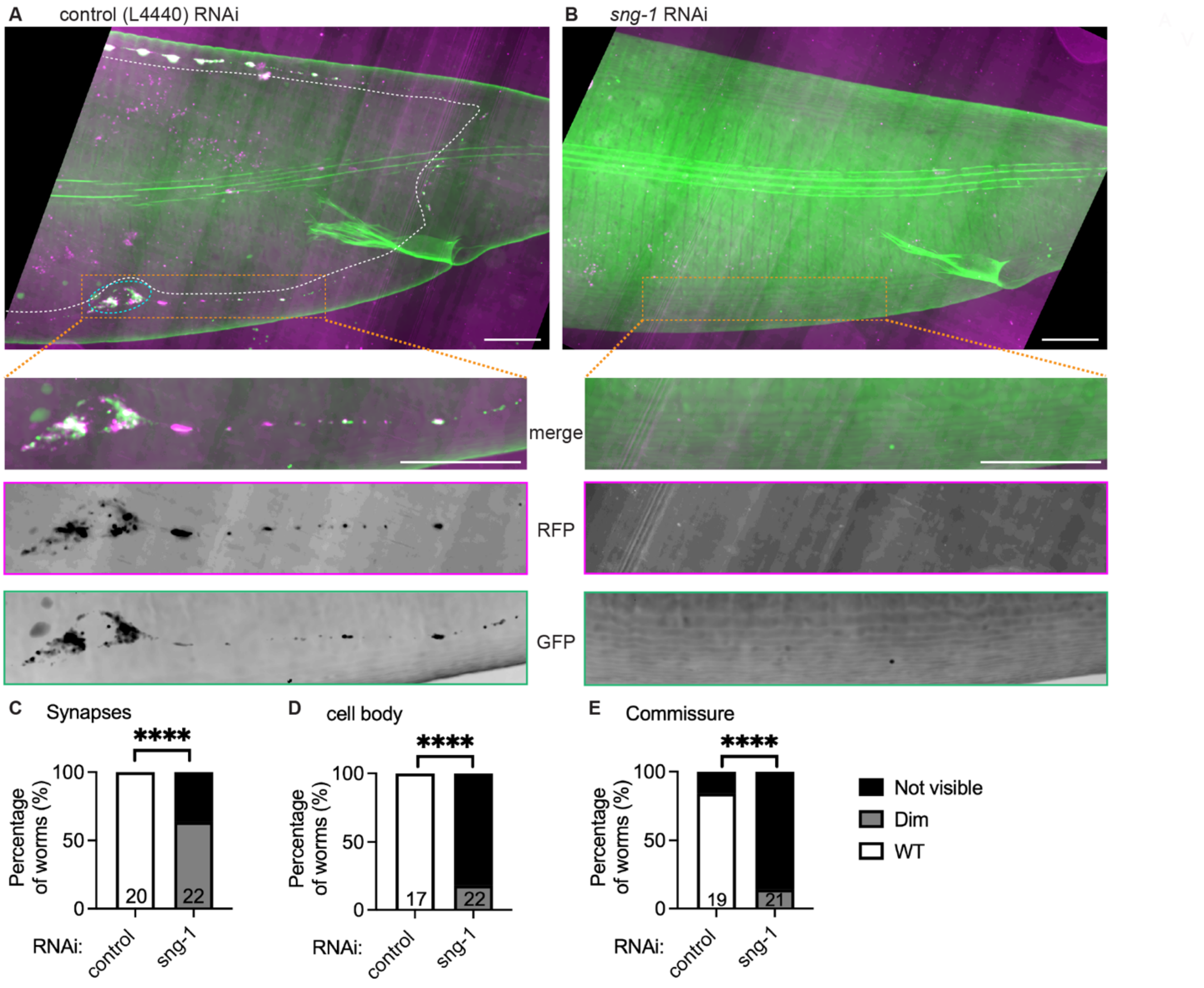

Figure S4. Validation that the RFP and GFP fluorescent puncta observed in the DA9 neuron are from SNG-1::ARGO. (A-B) Representative images of the DA neuron cell body (teal dashed oval), proximal axon, commissure, and proximal synaptic region taken with Super Resolution spinning disk confocal microscopy with deconvolution, similar to Figure 1I. For this experiment, a strain with the genotype *carIs1(DA9>Flp); eri-1; sng-1(syb3140car2[argo]) lin-15B* was generated – the *eri-1* and *lin-15B* alleles were added to provide efficient RNAi knockdown in the neurons. Animals were grown for 1.5 generations on feeding RNAi control (the empty vector L4440) (A) or against *sng-1* (B), and A3 animals were imaged with z-stacks through the entire animal. Maximum projections are shown. In (A), the white dashed line is adjacent to the neuron. In (B), no fluorescence from the neuron is discernable; the autofluorescence from the cuticle is more prominent in (B) compared to (A) because in (A), the maximum projection just includes z-slices in which the DA9 neuron is present, whereas in (B), because the neuron did not generate visible fluorescence, all z-slices were included. Scale = 10 μm. (C-D) Quantification of SNG-1: ARGO fluorescence in the synapses (C), the cell body (D), and the commissure and proximal axon (E) by RNAi treatment. For each compartment, the apparent fluorescence from SNG-1::ARGO in each animal was scored qualitatively as wild-type (WT), notably dim but

present (Dim), or not visible at all in the expected region (Not visible). The number of animals scored (n) is indicated in each bar. ****P<0.0001, Fisher's exact test.

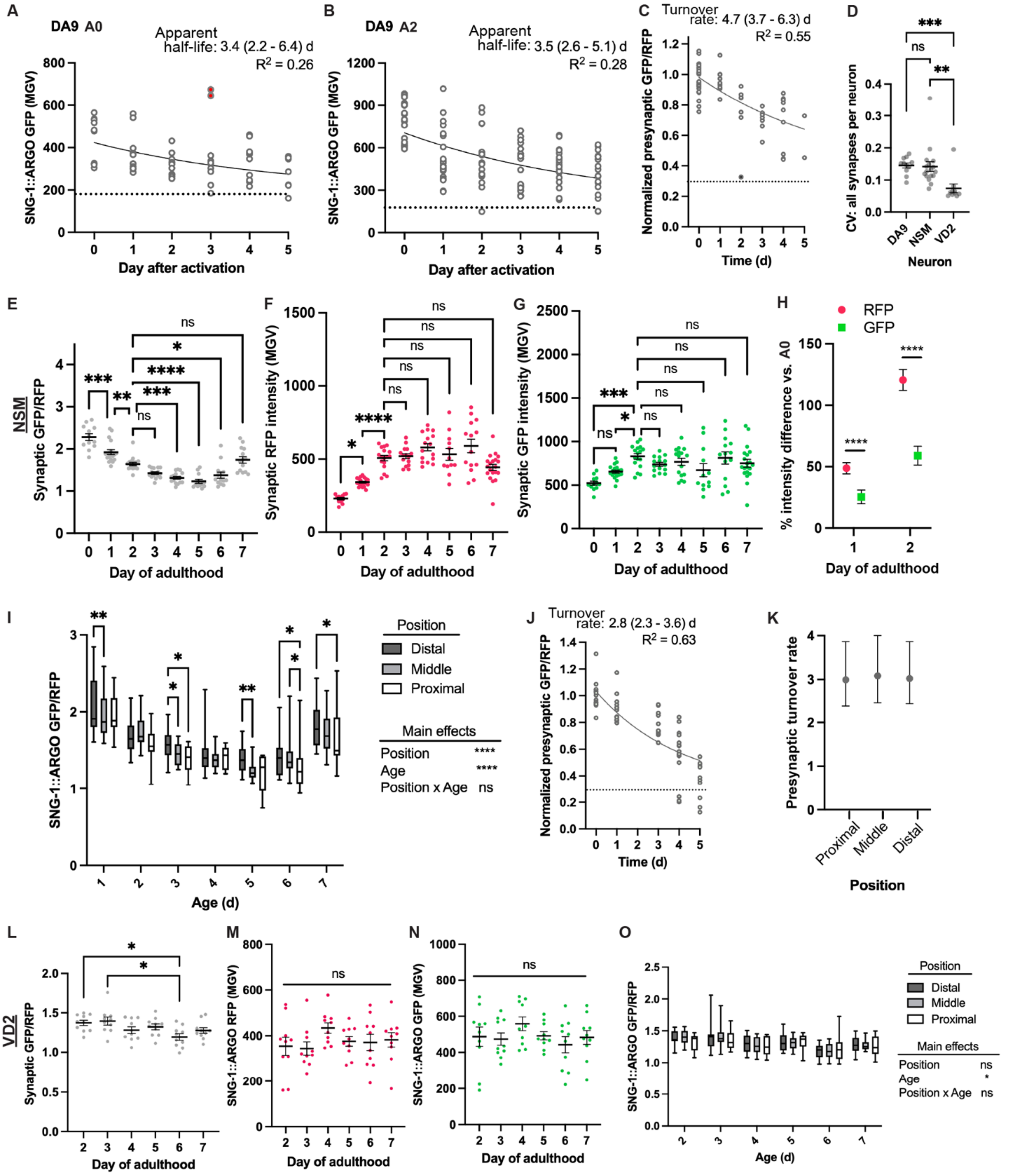

Figure S5. Additional analyses of SNG-1::ARGO steady-state fluorescence and turnover. (A-B) Quantification of SNG-1::ARGO apparent half-life in the DA9 neuron's presynapses using the mean GFP intensity at each presynapse. Each data point is the average of all the mean GFP values for one animal. Data were fit to one-phase exponential decay curves with the plateau set to the experimentally calculated plateau, which arose

from autofluorescence in the GFP channel. Filled circles indicate outlier data points that were excluded from the analysis. The data for these graphs came from the same experiments as those used to generate Figures 3B and 3E. Note that for the pulse at A0, the calculated SNG-1 half-life is longer using mean GFP, which only quantifies degradation (more precisely, the GFP quenching in acidic lysosomal compartments the precedes degradation), compared to using the GFP/RFP ratio (Figure 3E), which quantifies degradation plus synapse growth. By contrast, with the pulse at A2, the half-life calculated using mean GFP is indistinguishable from that calculated using the GFP/RFP ratio, though the 95% C.I. is substantially larger. (C) A turnover experiment in the DA9 neuron with the pulse at A2, wherein the animals were maintained at 25 °C after the pulse. These results are included for comparison to the *uba-1(it129ts)* turnover experiment; however, note that this is not the ideal comparison because the *uba-1(it129ts)* mutants were maintained at 25 °C from the L2 larval stage. (D) Comparison of intraneuronal variance between GFP/RFP ratio across individual presynapses. Each data point is the CV for all the presynaptic puncta in one neuron. (E-G) Average presynaptic RFP/GFP ratio (E), and the underlying intensity data for RFP (F) and GFP (G) from SNG-1::ARGO in the NSM neuron. Data points are each the average of all presynaptic puncta from a single neuron; bar shows mean + SEM. (H) Percent change in average presynaptic RFP and GFP intensity from A0 through A2. Plotted is mean ± SEM calculated from the data in F-G. (I) Steady-state SNG-1::ARGO presynaptic GFP/RFP by presynapse position along the axon in the NSM neuron. (J) One-phase exponential decay function fit to the data from an A2 SNG-1::ARGO turnover experiment in the NSM neuron; each data point shows the average presynaptic SNG-1::ARGO GFP/RFP ratio from all the presynpases in a single neuron. Dashed black line shows the experimentally calculated background, which comes from autofluorescence. Filled circles show outlier data points that were excluded from the curve calculation. (K) SNG-1::ARGO presynaptic half-life by proximal-distal position relative to the NSM neuron cell body (mean ± 95% C.I.). (L-N) Average presynaptic RFP/GFP ratio (L), and the underlying intensity data for RFP (M) and GFP (N) from SNG-1::ARGO in the VD2 neuron. Data points are each the average of all presynaptic puncta from a single neuron; bar shows mean ± SEM. (O) Steady-state SNG-1::ARGO presynaptic GFP/RFP by presynapse position along the axon in the VD2 neuron. ns: not significant, *P<0.05, **P<0.01, ***P<0.001, ****P<0.0001, one-way ANOVA or two-way ANOVA with Tukey post-test. For H and N, comparisons that were not significant are not shown.

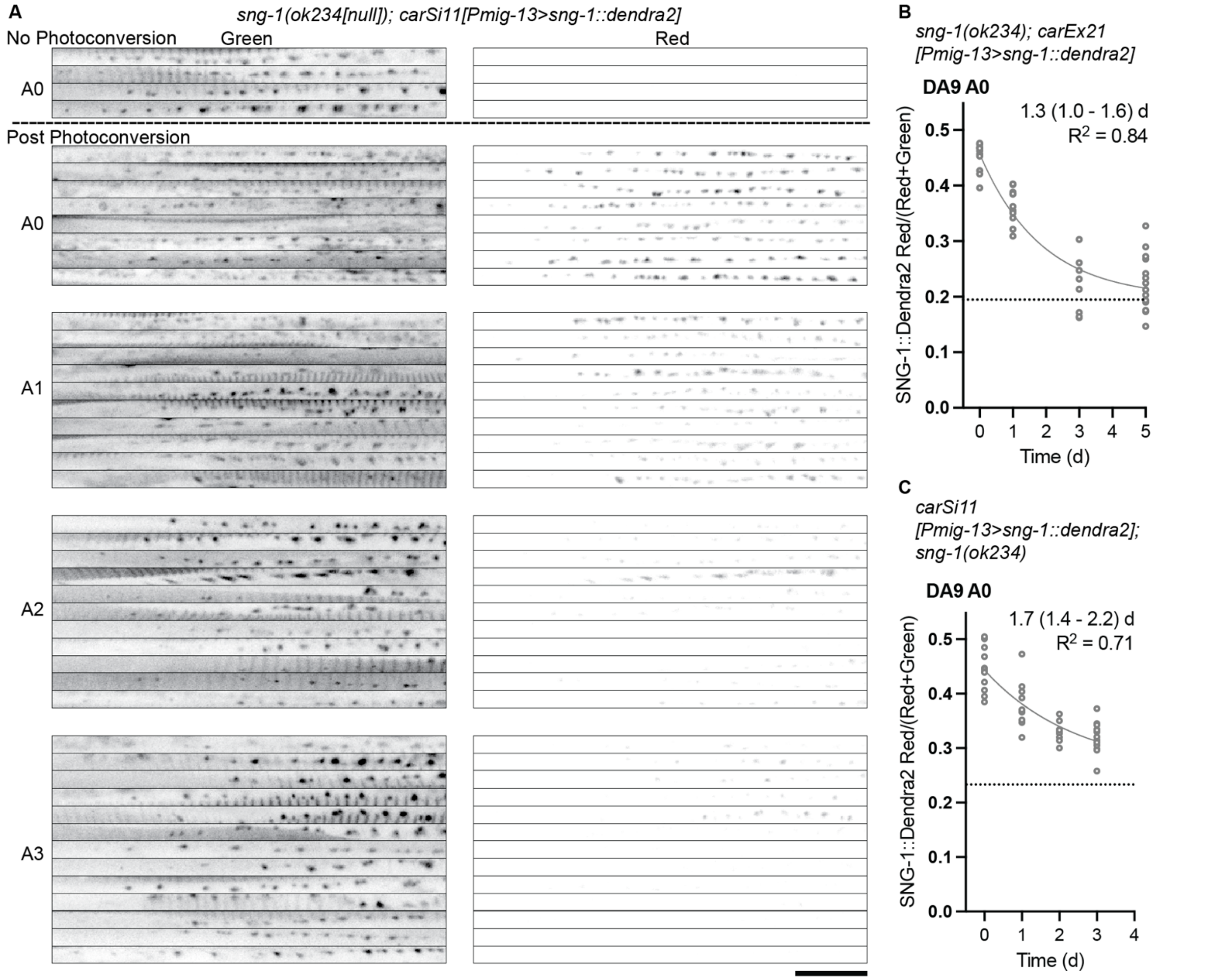

Figure S6. Analysis of presynaptic SNG-1::Dendra2 turnover in the DA9 neuron shows similar results compared with SNG-1::ARGO. (A) SNG-1::Dendra2 expressed in the DA9 neuron localizes to presynapses. The photoconversion, in which the whole animals were exposed as a cohort to 405 nm light, converted a substantial proportion of SNG-1::Dendra2 from the green-fluorescing variant to the red-fluorescing variant. Scale = 10 μm. (B-C) Turnover experiments using two different alleles of *sng-1::Dendra2* in the DA9 neuron give similar calculations of turnover rate.

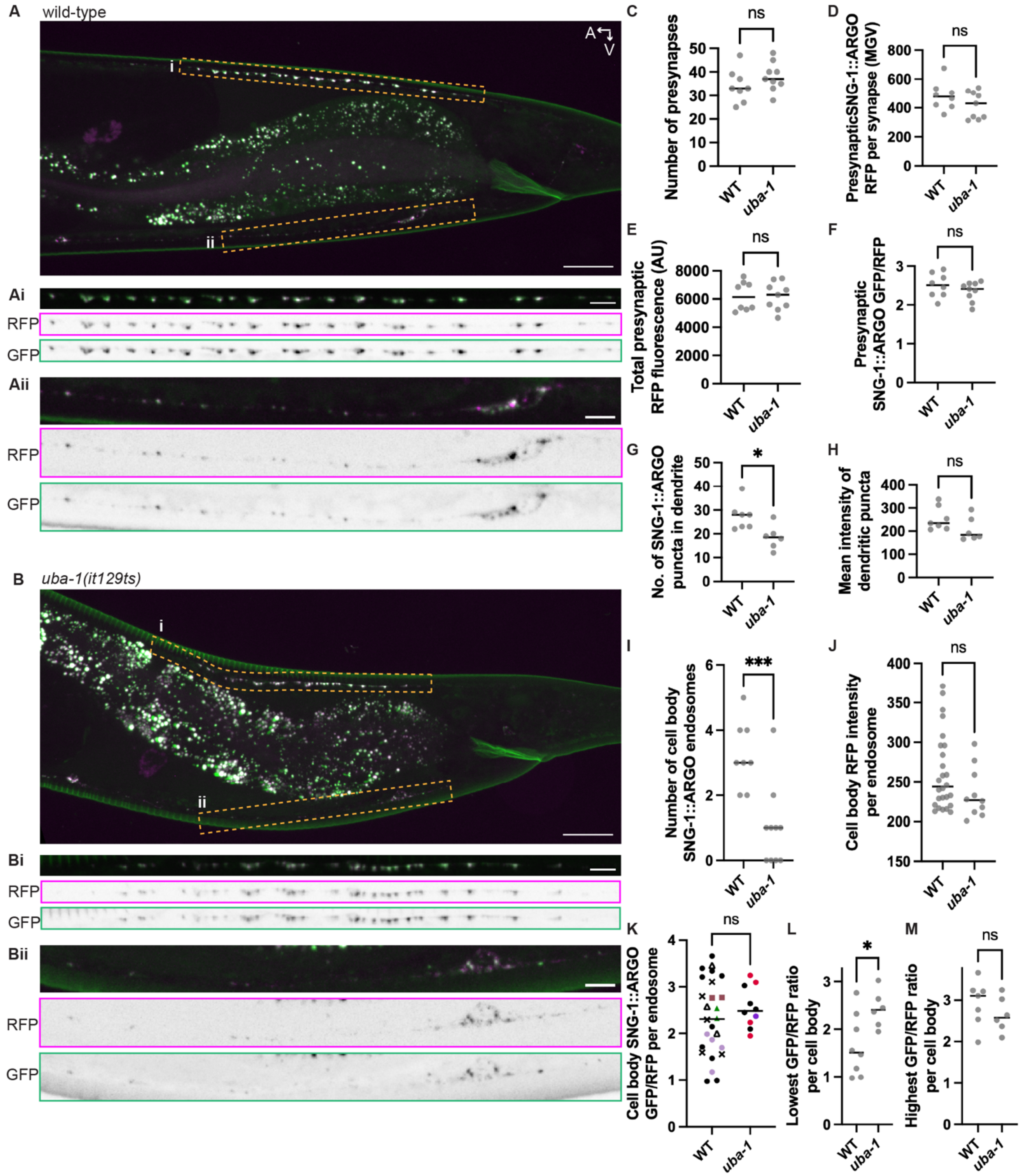

Figure S7. The *uba-1(it129ts)* mutant shows normal localization of SNG-1::ARGO at the presynapses but exhibits reduced SNG-1::ARGO fluorescence in the cell body and dendrite. Animals were shifted to the non-permissive temperature at the L2 larval stage and imaged at Day 2 of adulthood. (A-B) Representative images of SNG-1::ARGO in the DA9 neuron in wild-type (A) versus the *uba-1(it129ts)* mutant. Zoomed-in views show the presynaptic region (Ai, Bi) and the cell body and dendrite (Aii, Bii). Scale = 20 μm in zoomed-out views and 5 μm in zoomed-in views. (C-F) The *uba-1(it129ts)* mutant shows similar SNG-1::ARGO fluorescence to

wild-type in regard to number of presynapses (C), intensity per synapse (D), total presynaptic intensity (E), and presynaptic GFP/RFP ratio (F). (G-J) The *uba-1(it129ts)* mutant shows fewer dendritic SNG-1::ARGO endosomes (G) and fewer SNG-1::ARGO endosomes in the soma (I) with a statistically insignificant trend for decreased RFP fluorescence intensity per endosome (H, J). (K) No significant difference between the mean cell body SNG-1:: ARGO GFP/RFP ratio per endosome, though wild-type shows a trend for a larger range of ratios (P=0.06, F test). (L-M) In the *uba-1(it129ts)* mutant, the soma's lowest endosomal SNG-1::ARGO GFP/RFP ratio is higher than it is in wild-type (L), though the soma's highest endosomal GFP/RFP ratio is not significantly different from wild-type (M). ns: not significant, *P<0.05, ***P<0.001, (C-I, L-M) Welch's two-tailed t-test (chosen because it does not assume equal variance), (J-K) Two-tailed Mann-Whitney test (chosen because the distribution of the data appears non-parametric).

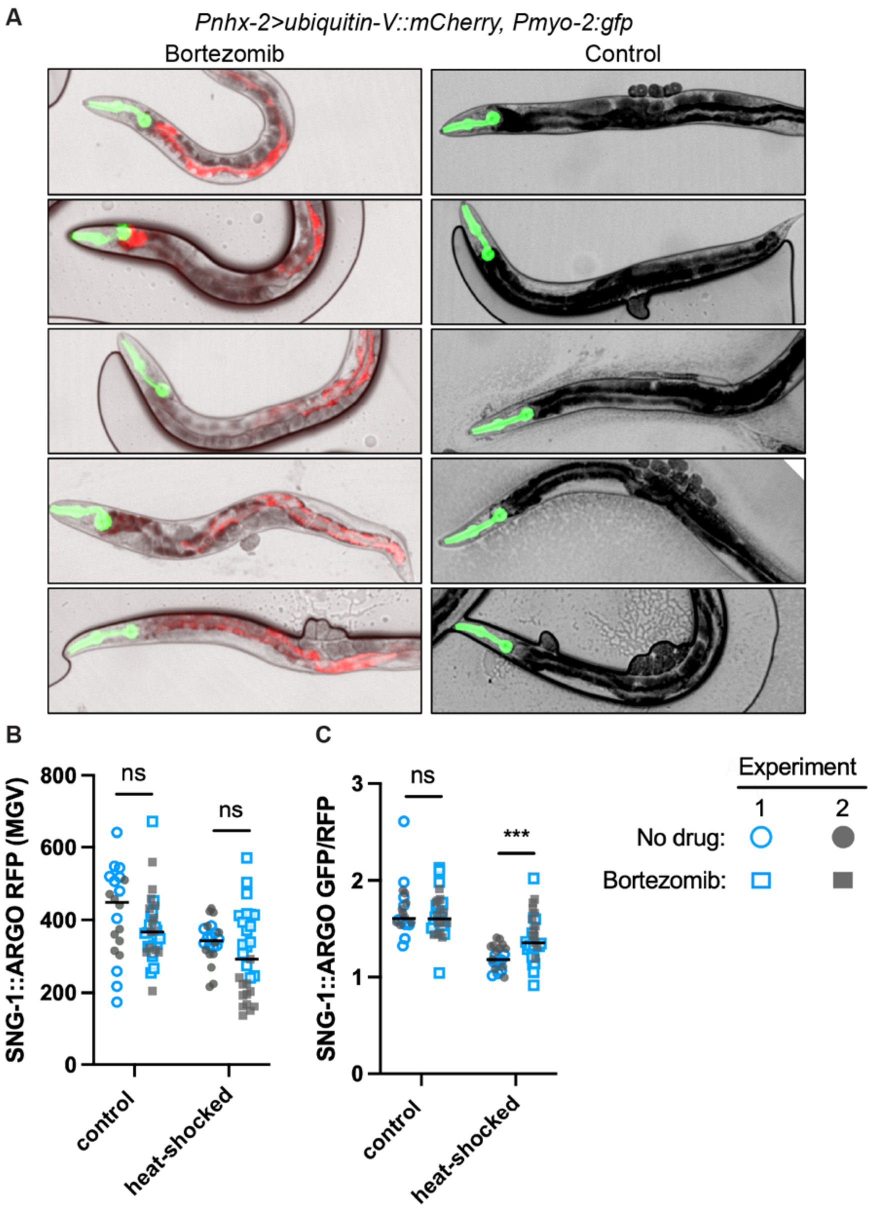

Figure S8. Treatment with proteasome inhibitor bortezomib delays SNG-1::ARGO turnover from presynapses. (A) Representative images of *vkEx1243(Pnhx-2>ubiquitin-V::mCherry, Pmyo-2>gfp)* animals treated with Bortezomib versus control. In this strain, the Ubiquitin-V::mCherry is normally expressed in the intestine but rapidly degraded by the proteasome; its degradation is blocked by bortezomib. (B-C) Quantification from two independent experiments in which animals were chronically treated with bortezomib from A0, pulsed (heat-shocked) versus not pulsed at A2, and imaged at A5. Bortezomib-treated animals showed no obvious change in SNG-1::ARGO accumulation at presynapses, measured by mean presynaptic RFP intensity (A), and no apparent change in presynaptic steady-state SNG-1::ARGO GFP/RFP ratio (B), but increased presynaptic SNG-1::ARGO GFP/RFP ratio after the heat-shock pulse, indicative of slower turnover (B). Ns: not significant, ***$P<0.001$, linear mixed model with drug (bortezomib versus none) and treatment (heat shock-pulse versus no pulse) as fixed effects and experiment as a random effect, followed by Tukey-corrected pairwise comparisons.

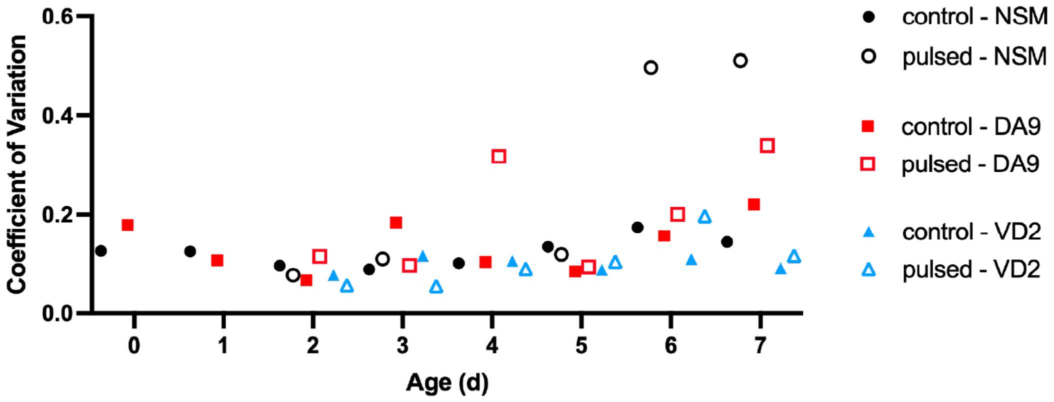

Figure S9. Interneuronal Coefficient of Variation (CV) for each neuron identity during steady-state imaging and the turnover experiment. The analyses were performed on the same datasets used throughout the manuscript for steady-state imaging and turnover with the pulse at A2. As there is one dataset per neuron identity, there is one CV per neuron/age/treatment. This is therefore a qualitative assessment of SNG-1::ARGO specificity and efficacy by neuron. It complements the results in Table 1 in that it is better designed to detect instances wherein a single allele of *gfp* is removed rather than both copies. Note that the VD2 neuron does not show higher CVs at any age compared to the DA9 and NSM neurons, so the two-phase turnover in the VD2 neuron is not due to unselective of ineffective *gfp* removal.

cDNA sng-1 syb3140car2 (1619 bp)

```
CCATGCAACAACCACCATCAAACCCATAtACTCAaTCgGAAGGATATGGTTATGGAAGTTCCTATTCTCTAGAAAGTATAGGA
GGTACGTTGTTGGTGGTAGTTTGGGTATaTGAGTtAGcCTTCCTATACCAATACCTTCAAGGATAAGAGATCTTTCATATCCT
 2 4 6 8 10 12 14 16 18 20 22 24 26
M Q Q P P S N P Y T Q S E G Y G Y G S S Y S L E S I G
sng-1:Argo
sng-1 C-term  FRT
10 20 30 40 50 60 70 80
```

```
ACTTCAGTCTCCAAGGGAGAGGAGCTCATCAAGGAGAACATGCACATGAAGCTCTACATGGAGGGAACCGTCAACAACCACCA
TGAAGTCAGAGGTTCCCTCTCCTCGAGTAGTTCCTCTTGTACGTGTACTTCGAGATGTACCTCCCTTGGCAGTTGTTGGTGGT
28 30 32 34 36 38 40 42 44 46 48 50 52 54
T S V S K G E E L I K E N M H M K L Y M E G T V N N H H
sng-1:Argo
tagRFP
90 100 110 120 130 140 150 160
```

```
CTTCAAGTGCACCTCCGAGGGAGAGGGAAAGCCATACGAGGGAACCCAAACCATGCGTATCAAGTCGTCGAGGGAGGACCACT
GAAGTTCACGTGGAGGCTCCCTCTCCCTTTCGGTATGCTCCCTTGGGTTTGGTACGCATAGTTCAGCAGCTCCCTCCTGGTGA
56 58 60 62 64 66 68 70 72 74 76 78 80 82
F K C T S E G E G K P Y E G T Q T M R I K S S R E D H
sng-1:Argo
tagRFP
170 180 190 200 210 220 230 240
```

```
CCCATTCGCCTTCGACATCCTCGCCACCTCCTTCATGTACGGATCCCGTACCTTCATCAACCACACCCAAGGAATCCCAGACT
GGGTAAGCGGAAGCTGTAGGAGCGGTGGAGGAAGTACATGCCTAGGGCATGGAAGTAGTTGGTGTGGGTTCCTTAGGGTCTGA
84 86 88 90 92 94 96 98 100 102 104 106 108 110
S H S P S T S S P P P S C T D P V P S S T T P K E S Q T
sng-1:Argo
tagRFP
250 260 270 280 290 300 310 320 330
```

```
TCTTCAAGCAATCCTTCCCAGAGGGATTCACCTGGGAGCGTGTCACCACCTACGAGGACGGAGGAGTCCTCACCGCCACCCAA
AGAAGTTCGTTAGGAAGGGTCTCCCTAAGTGGACCCTCGCACAGTGGTGGATGCTCCTGCCTCCTCAGGAGTGGCGGTGGGTT
112 114 116 118 120 122 124 126 128 130 132 134 136 138
S S S N P S Q R D S P G S V S P P T R T E E S S P P P K
sng-1:Argo
tagRFP
340 350 360 370 380 390 400 410
```

GACACCTCCCTCCAAGACGGATGCCTCATCTACAACGTCAATCCGTGGAGTCAACTTCCCATCCAACGGACCAGTCATGCAAA
CTGTGGAGGGAGGTTCTGCCTACGGAGTAGATGTTGCAGTTAGGCACCTCAGTTGAAGGGTAGGTTGCCTGGTCAGTACGTTT
140 142 144 146 148 150 152 154 156 158 160 162 164
T P P S K T D A S S T T S I R G V N F P S N G P V M Q
sng-1:Argo
tagRFP
420 430 440 450 460 470 480 490

AGAAGACCCTCGGATGGGAGGCCAACACCGAGATGCTCTACCCAGCCGACGGAGGACTcGAGGGACGTACCGACATGGCCCTC
TCTTCTGGGAGCCTACCCTCCGGTTGTGGCTCTACGAGATGGGTCGGCTGCCTCCTGAgCTCCCTGCATGGCTGTACCGGGAG
166 168 170 172 174 176 178 180 182 184 186 188 190 192
K K T L G W E A N T E M L Y P A D G G L E G R T D M A L
sng-1:Argo
tagRFP
500 510 520 530 540 550 560 570 580

AAGCTCGTCGGAGGAGGACACCTCATCTGCAACTTCAAGACCACCTACCGTTCCAAGCCAGCCAAGAACCTCAAGATGCCAGG
TTCGAGCAGCCTCCTCCTGTGGAGTAGACGTTGAAGTTCTGGTGGATGGCAAGGTTCGGTCGGTTCTTGGAGTTCTACGGTCC
194 196 198 200 202 204 206 208 210 212 214 216 218 220
K L V G G G H L I C N F K T T Y R S K P A K N L K M P G
sng-1:Argo
tagRFP
590 600 610 620 630 640 650 660

AGTCTACTACGTCGACCACCGTCTcGAGCGTATCAAGGAGGCCGACAAGGAGACcTACGTCGAGCAACACGAGGTCGCCGTCG
TCAGATGATGCAGCTGGTGGCAGAgCTCGCATAGTTCCTCCGGCTGTTCCTCTGgATGCAGCTCGTTGTGCTCCAGCGGCAGC
222 224 226 228 230 232 234 236 238 240 242 244 246 248
V Y Y V D H R L E R I K E A D K E T Y V E Q H E V A V
sng-1:Argo
tagRFP
670 680 690 700 710 720 730 740

CCCGTTACTGCGACCTCCCATCCAAGCTCGGACACAAGCTCAACGgTatggaTgaActGtaTaaGGGTTCTGGGAGTGGAAGC
GGGCAATGACGCTGGAGGGTAGGTTCGAGCCTGTGTTCGAGTTGCcAtacctActTgaCatAttCCCAAGACCCTCACCTTCG
250 252 254 256 258 260 262 264 266 268 270 272 274 276
A R Y C D L P S K L G H K L N G M D E L Y K G S G S G S
sng-1:Argo
tagRFP GSGSGSGS linker
750 760 770 780 790 800 810 820 830

```
GGCTCTATGAGTAAAGGAGAAGAACTTTTCACTGGAGTTGTCCCAATTCTTGTTGAATTAGATGGTGATGTTAATGGGCACAA
CCGAGATACTCATTTCCTCTTCTTGAAAAGTGACCTCAACAGGGTTAAGAACAACTTAATCTACCACTACAATTACCCGTGTT
   278  280  282  284  286  288  290  292  294  296  298  300  302  304
 G  S  M  S  K  G  E  E  L  F  T  G  V  V  P  I  L  V  E  L  D  G  D  V  N  G  H  K
sng-1:Argo
GFP
840  850  860  870  880  890  900  910
```

```
ATTTTCTGTCAGTGGAGAGGGTGAAGGTGATGCAACATACGGAAAACTTACCCTTAAATTTATTTGCACTACTGGAAAACTAC
TAAAAGACAGTCACCTCTCCCACTTCCACTACGTTGTATGCCTTTTGAATGGGAATTTAAATAAACGTGATGACCTTTTGATG
   306  308  310  312  314  316  318  320  322  324  326  328  330
 F  S  V  S  G  E  G  E  G  D  A  T  Y  G  K  L  T  L  K  F  I  C  T  T  G  K  L
sng-1:Argo
GFP
920  930  940  950  960  970  980  990
```

```
CTGTTCCATGGCCAACACTTGTCACTACTTTCTGTTATGGTGTTCAATGCTTCTCGAGATACCCAGATCATATGAAACGGCAT
GACAAGGTACCGGTTGTGAACAGTGATGAAAGACAATACCACAAGTTACGAAGAGCTCTATGGGTCTAGTATACTTTGCCGTA
332  334  336  338  340  342  344  346  348  350  352  354  356  358
 P  V  P  W  P  T  L  V  T  T  F  C  Y  G  V  Q  C  F  S  R  Y  P  D  H  M  K  R  H
sng-1:Argo
GFP
1,000  1,010  1,020  1,030  1,040  1,050  1,060  1,070
```

```
GACTTTTTCAAGAGTGCCATGCCCGAAGGTTATGTACAGGAAAGAACTATATTTTTCAAAGATGACGGGAACTACAAGACACG
CTGAAAAAGTTCTCACGGTACGGGCTTCCAATACATGTCCTTTCTTGATATAAAAAGTTTCTACTGCCCTTGATGTTCTGTGC
360  362  364  366  368  370  372  374  376  378  380  382  384  386
 D  F  F  K  S  A  M  P  E  G  Y  V  Q  E  R  T  I  F  F  K  D  D  G  N  Y  K  T  R
sng-1:Argo
GFP
1,080  1,090  1,100  1,110  1,120  1,130  1,140  1,150  1,160
```

```
TGCTGAAGTCAAGTTTGAAGGTGATACCCTTGTTAATAGAATCGAGTTAAAAGGTATTGATTTTAAAGAAGATGGAAACATTC
ACGACTTCAGTTCAAACTTCCACTATGGGAACAATTATCTTAGCTCAATTTTCCATAACTAAAATTTCTTCTACCTTTGTAAG
  388  390  392  394  396  398  400  402  404  406  408  410  412  414
 A  E  V  K  F  E  G  D  T  L  V  N  R  I  E  L  K  G  I  D  F  K  E  D  G  N  I
sng-1:Argo
GFP
1,170  1,180  1,190  1,200  1,210  1,220  1,230  1,240
```

```
TTGGACACAAATTGGAATACAACTaTAACTCACACAATGTATACATCATGGCAGACAAACAAAAGAATGGAATCAAAGtTAAC
AACCTGTGTTTAACCTTATGTTGAtATTGAGTGTGTTACATATGTAGTACCGTCTGTTTGTTTTCTTACCTTAGTTTCaATTG
416 418 420 422 424 426 428 430 432 434 436 438 440 442
L G H K L E Y N Y N S H N V Y I M A D K Q K N G I K V N
sng-1:Argo
GFP
1,250 1,260 1,270 1,280 1,290 1,300 1,310 1,320
```

```
TTCAAAATTAGACACAACATTGAAGATGGAAGCGTTCAACTAGCAGACCATTATCAACAAAATACTCCAATTGGCGATGGCCC
AAGTTTTAATCTGTGTTGTAACTTCTACCTTCGCAAGTTGATCGTCTGGTAATAGTTGTTTTATGAGGTTAACCGCTACCGGG
444 446 448 450 452 454 456 458 460 462 464 466 468 470
F K I R H N I E D G S V Q L A D H Y Q Q N T P I G D G P
sng-1:Argo
GFP
1,330 1,340 1,350 1,360 1,370 1,380 1,390 1,400 1,410
```

```
TGTCCTTTTACCAGACAACCATTACCTGtCCACACAATCTGCCCTTTCGAAAGATCCCAACGAAAAGAGAGACCACATGGTCC
ACAGGAAAATGGTCTGTTGGTAATGGACaGGTGTGTTAGACGGGAAAGCTTTCTAGGGTTGCTTTTCTCTCTGGTGTACCAGG
472 474 476 478 480 482 484 486 488 490 492 494 496
V L L P D N H Y L S T Q S A L S K D P N E K R D H M V
sng-1:Argo
GFP
1,420 1,430 1,440 1,450 1,460 1,470 1,480 1,490
```

```
TTcTTGAGTTTGTAACAGCTGCTGGGATTACACATGGCATGGATGAACTATACAAAATAACTTCGTATAGCATACATTATACG
AAgAACTCAAACATTGTCGACGACCCTAATGTGTACCGTACCTACTTGATATGTTTTATTGAAGCATATCGTATGTAATATGC
498 500 502 504 506 508 510 512 514 516 518 520 522 524
L L E F V T A A G I T H G M D E L Y K I T S Y S I H Y T
sng-1:Argo
GFP LoxP
1,500 1,510 1,520 1,530 1,540 1,550 1,560 1,570
```

```
AAGTTATAGtttttagccaaacaaaaaaaaacacatacaaa
TTCAATATCaaaaatcggtttgtttttttttgtgtatgttt
526 528
K L *
sng-1...
LoxP
1,580 1,590 1,600 1,610
```

syb3140car2 recombined (891 bp)

```
CCATGCAACAACCACCATCAAACCCATAtACTCAaTCgGAAGGATATGGTTATGGAAGTTCCTATTCTCTAGAAAGTATAGGAACTTCAGTCT
GGTACGTTGTTGGTGGTAGTTTGGGTATaTGAGTtAGcCTTCCTATACCAATACCTTCAAGGATAAGAGATCTTTCATATCCTTGAAGTCAGA
   2    4    6    8    10   12   14   16   18   20   22   24   26   28   30
M  Q  Q  P  P  S  N  P  Y  T  Q  S  E  G  Y  G  Y  G  S  S  Y  S  L  E  S  I  G  T  S  V
Translation
sng-1 C-term    FRT    »
10  20  30  40  50  60  70  80  90
```

```
CCAAGGGAGAGGAGCTCATCAAGGAGAACATGCACATGAAGCTCTACATGGAGGGAACCGTCAACAACCACCACTTCAAGTGCACCTCCGAGG
GGTTCCCTCTCCTCGAGTAGTTCCTCTTGTACGTGTACTTCGAGATGTACCTCCCTTGGCAGTTGTTGGTGGTGAAGTTCACGTGGAGGCTCC
 32   34   36   38   40   42   44   46   48   50   52   54   56   58   60
S K  G  E  E  L  I  K  E  N  M  H  M  K  L  Y  M  E  G  T  V  N  N  H  H  F  K  C  T  S  E
Translation
tagRFP
100  110  120  130  140  150  160  170  180
```

```
GAGAGGGAAAGCCATACGAGGGAACCCAAACCATGCGTATCAAGGTCGTCGAGGGAGGACCACTCCCATTCGCCTTCGACATCCTCGCCACCT
CTCTCCCTTTCGGTATGCTCCCTTGGGTTTGGTACGCATAGTTCCAGCAGCTCCCTCCTGGTGAGGGTAAGCGGAAGCTGTAGGAGCGGTGGA
62   64   66   68   70   72   74   76   78   80   82   84   86   88   90   92
G E  G  K  P  Y  E  G  T  Q  T  M  R  I  K  V  V  E  G  G  P  L  P  F  A  F  D  I  L  A  T
Translation
tagRFP
190  200  210  220  230  240  250  260  270
```

```
CCTTCATGTACGGATCCCGTACCTTCATCAACCACACCCAAGGAATCCCAGACTTCTTCAAGCAATCCTTCCCAGAGGGATTCACCTGGGAGC
GGAAGTACATGCCTAGGGCATGGAAGTAGTTGGTGTGGGTTCCTTAGGGTCTGAAGAAGTTCGTTAGGAAGGGTCTCCCTAAGTGGACCCTCG
 94   96   98   100  102  104  106  108  110  112  114  116  118  120  122
S F  M  Y  G  S  R  T  F  I  N  H  T  Q  G  I  P  D  F  F  K  Q  S  F  P  E  G  F  T  W  E
Translation
tagRFP
280  290  300  310  320  330  340  350  360  370
```

```
GTGTCACCACCTACGAGGACGGAGGAGTCCTCACCGCCACCCAAGACACCTCCCTCCAAGACGGATGCCTCATCTACAACGTCAAGATCCGTG
CACAGTGGTGGATGCTCCTGCCTCCTCAGGAGTGGCGGTGGGTTCTGTGGAGGGAGGTTCTGCCTACGGAGTAGATGTTGCAGTTCTAGGCAC
124  126  128  130  132  134  136  138  140  142  144  146  148  150  152  154
R V  T  T  Y  E  D  G  G  V  L  T  A  T  Q  D  T  S  L  Q  D  G  C  L  I  Y  N  V  K  I  R
Translation
tagRFP
380  390  400  410  420  430  440  450  460
```

GAGTCAACTTCCCATCCAACGGACCAGTCATGCAAAAGAAGACCCTCGGATGGGAGGCCAACACCGAGATGCTCTACCCAGCCGACGGAGGAC
CTCAGTTGAAGGGTAGGTTGCCTGGTCAGTACGTTTTCTTCTGGGAGCCTACCCTCCGGTTGTGGCTCTACGAGATGGGTCGGCTGCCTCCTG
156 158 160 162 164 166 168 170 172 174 176 178 180 182 184
G V N F P S N G P V M Q K K T L G W E A N T E M L Y P A D G G
Translation
tagRFP
470 480 490 500 510 520 530 540 550

TcGAGGGACGTACCGACATGGCCCTCAAGCTCGTCGGAGGAGGACACCTCATCTGCAACTTCAAGACCACCTACCGTTCCAAGAAGCCAGCCA
AgCTCCCTGCATGGCTGTACCGGGAGTTCGAGCAGCCTCCTCCTGTGGAGTAGACGTTGAAGTTCTGGTGGATGGCAAGGTTCTTCGGTCGGT
186 188 190 192 194 196 198 200 202 204 206 208 210 212 214 216
L E G R T D M A L K L V G G G H L I C N F K T T Y R S K K P A
Translation
tagRFP
560 570 580 590 600 610 620 630 640 650

AGAACCTCAAGATGCCAGGAGTCTACTACGTCGACCACCGTCTcGAGCGTATCAAGGAGGCCGACAAGGAGACcTACGTCGAGCAACACGAGG
TCTTGGAGTTCTACGGTCCTCAGATGATGCAGCTGGTGGCAGAgCTCGCATAGTTCCTCCGGCTGTTCCTCTGgATGCAGCTCGTTGTGCTCC
218 220 222 224 226 228 230 232 234 236 238 240 242 244 246
K N L K M P G V Y Y V D H R L E R I K E A D K E T Y V E Q H E
Translation
tagRFP
660 670 680 690 700 710 720 730 740

TCGCCGTCGCCCGTTACTGCGACCTCCCATCCAAGCTCGGACACAAGCTCAACGgTatggaTgaActGtaTaaGgtaagtttaaacatgattt
AGCGGCAGCGGGCAATGACGCTGGAGGGTAGGTTCGAGCCTGTGTTCGAGTTGCcAtacctActTgaCatAttCcattcaaatttgtactaaa
248 250 252 254 256 258 260 262 264 266 268 270 272 274 276 278
V A V A R Y C D L P S K L G H K L N G M D E L Y K V S L N M I
Translation
tagRFP
750 760 770 780 790 800 810 820 830

tactaacATAACTTCGTATAGCATACATTATACGAAGTTATAGttttttagcca
atgattgTATTGAAGCATATCGTATGTAATATGCTTCAATATCaaaaaatcggt
280 282
L L T *
Translation
840 850 860 870 880 890
